# Information Certainty Determines Social and Private Information Use in Ants

**DOI:** 10.1038/srep43607

**Published:** 2017-03-03

**Authors:** Nathalie Stroeymeyt, Martin Giurfa, Nigel R. Franks

**Affiliations:** 1 School of Biological Sciences, University of Bristol, Life Sciences Building, 24 Tyndall Avenue, Bristol, BS8 1TQ, United Kingdom; 2 Research Center on Animal Cognition, Center for Integrative Biology, University of Toulouse, CNRS, UPS 118 route de Narbonne, 31062 Toulouse cedex 9, France; 3 Département d’Ecologie et d’Evolution, Biophore, Quartier UNIL-Sorge, Université de Lausanne, Lausanne, CH-1015, Switzerland

## Abstract

Decision-making in uncertain environments requires animals to evaluate, contrast and integrate various information sources to choose appropriate actions. In consensus-making groups, quorum responses are commonly used to combine private and social information, leading to both robust and flexible decisions. Here we show that in house-hunting ant colonies, individuals fine-tune the parameters of their quorum responses depending on their private knowledge: informed ants evaluating a familiar new nest rely relatively more on social than private information when the certainty of their private information is low, and vice versa. This indicates that the ants follow a highly sophisticated ‘copy-when-uncertain’ social learning strategy similar to that observed in a few vertebrate species. Using simulations, we further show that this strategy improves colony performance during emigrations and confers well-informed individuals more weight in the decision process, thus representing a novel mechanism for the emergence of leadership in collective decision-making.

When choosing between alternative mates, habitats, or food sources, animals usually only have partial, noisy information about the value of the options. Robust strategies for processing and integrating diverse sources of information are therefore central to decision-making in uncertain environments. In particular, individuals need to find an appropriate balance between (i) acquiring new information through private exploration, (ii) using previously acquired private information, and (iii) using social information acquired by other individuals. Each of these information sources is associated with different costs and benefits^1^,^2^,^3^. For example, existing private information incurs low costs and is usually reliable, but it can become outdated and it does not allow individuals to track environmental changes^4^,^5^,^6^,^7^,^8^. On the other hand, social information is cheaper to acquire than new private information because of the costs and risks associated with exploration. However, it is also less reliable, because animals make mistakes when interpreting social cues and may copy poorly informed individuals, leading to false informational cascades^2^,^9^,^10^. Theory therefore predicts that animals should use flexible social learning strategies: social information should only be used in specific conditions, for example when private information is costly to acquire (‘copy-when-costly’), when it is absent, unreliable or outdated (‘copy-when-uncertain’), or when it leads to unproductive decisions (‘copy-when-dissatisfied’)^1^,^2^,^3^.

In animal groups making consensus decisions, the optimal balance between social and private information is further complicated by the central role of information sharing in the decision process^11^. Theory predicts that pooling information across an increasing number of group members should increase collective accuracy (‘wisdom of crowds’ effect), but this depends on individuals gathering information independently of one another^10^,^12^,^13^,^14^,^15^,^16^,^17^,^18^,^19^,^20^,^21^,^22^,^23^. There is, therefore, an inherent paradox between the need for group members to rely on private information in order to avoid systematic biases and false informational cascades, and the need to exchange social information in order to reach an accurate consensus. In addition, individuals in a group usually vary in the quality of their private information. Theory predicts that the accuracy of collective decisions could be further increased if well-informed individuals have a disproportionately high influence on the decision process^12^. However, empirical studies investigating the relationship between private information, individual behaviour and collective patterns in real animal groups are still lacking. Here, we show experimentally that the certainty of individual knowledge influences the primary source of information used by decision-makers in a multi-choice, decentralised consensus decision: nest site selection by the ant *Temnothorax albipennis*. This is the first experimental demonstration of a ‘copy-when-uncertain’ social learning strategy in invertebrates^2^,^3^,^4^,^24^. We further show that foregoing social information in favour of private information allows the best-informed individuals to emerge as leaders, thus constituting a novel mechanism for emergent leadership in self-organising groups.

*Temnothorax* colonies occupy fragile natural nest sites such as hollow acorns, rotten twigs or rock crevices, and often have to move to a new nest (‘emigrate’) when conditions deteriorate. During emigrations, colonies are able to select the best among several available sites^25^ through distributed decision-making mechanisms. When their nest has been damaged, scout workers look for suitable nest sites. Upon finding a candidate site, scouts evaluate it, and if they deem it suitable, they recruit nestmates to it by tandem running^26^. Recruits then evaluate the site independently and may in turn initiate recruitment. The recruitment rate is greater for high quality nest sites^26^, so the nest population grows faster in better sites. Scouts then switch from slow recruitment by tandem running to fast carrying of brood and nestmates following a typical ‘quorum response’, in which the probability of switching to transport is a sharply non-linear function of the number of workers already present inside the new nest^27^,^28^,^29^. Because the nest population increases faster in better sites, scouts start carrying to those sites earlier, which ensures that all or most of the colony is transported to the better option^29^. The quorum response provides an adaptive strategy for integrating private and social information: individuals initially acquire new private information independently when evaluating the candidate site, then rely on social information once the number of nestmates exceeds a quorum threshold^27^,^28^. This greatly reduces the risk that an initially incorrect evaluation is amplified by chance, and increases overall accuracy through opinion pooling^9^,^12^,^13^,^18^,^27^,^29^. Additionally, recent studies showed that *Temnothorax* ants can also use existing private information during emigrations: informed workers that have previously visited suitable nest sites use their memory to navigate to these sites efficiently in later emigrations, thus increasing emigration speed and decision accuracy^30^,^31^. Here we show experimentally that memory also affects the relative use of private vs. social information during the nest evaluation process. Using a dataset from a previous study^30^, we further investigated the relationship between the certainty of private information and the degree to which informed workers rely on private vs. social information when evaluating familiar nests. Following previous experimental studies of social learning in ants and chimpanzees^8^,^32^, we used the number of visits to the familiar nest site prior to emigration as a measure for the certainty of private information. Our results are consistent with a sophisticated, graded ‘copy-when-uncertain’ strategy, whereby individuals increasingly prioritise social over private information as the certainty of their private information decreases. Finally, we performed simulations to determine how this strategy might affect colony performance during emigrations.

## Results

### The use of memory in familiar nest evaluation (‘memory experiment’)

**Figure 1:**
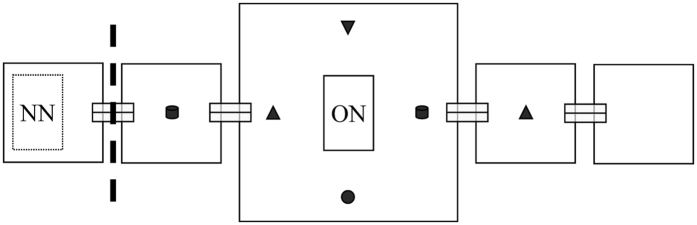
Experimental arena. Top view of the experimental arena consisting of a central large Petri dish (22 × 22 × 2.2 cm), 2 intermediate and 2 peripheral small Petri dishes (10 × 10 × 1.7 cm). Adjacent dishes were connected by tunnels made of 2 spectrometry cuvettes (12.5 × 12.5 × 49 mm) positioned side by side and whose bases were cut off. Conspicuous landmarks (black shapes) were used to help ants orient inside the arena. Colonies housed in their old nest (ON) were positioned in the middle of the central dish and left to explore the arena for one week. We then destroyed the old nest to induce colonies to emigrate to a new nest (NN) pseudo-randomly positioned in one of the two peripheral dishes. The new nest was identical to their old nest and either familiar (i.e. present throughout the exploration period) or unfamiliar (i.e. introduced in the arena just before emigration). The dashed line represents the position of acetate sliding doors fitted through the tunnels leading from the intermediary dish to the peripheral dish containing the new nests. These doors were manually lifted or lowered during emigration in order to restrict access to the new nest (see Methods for details).

We first investigated the effect of three types of information on the evaluation of familiar nest sites during forced emigrations: physical contacts with nestmates (nest population), chemical cues, and memory. As in previous studies, we found that assessment time (time between the first ant entering the new nest and the first transport of an adult or brood item into the new nest) was lower for familiar than unfamiliar new nests. This difference was not due to familiar nests reaching a fixed quorum threshold earlier than unfamiliar nests. Instead, scouts initiated transport at lower nest populations in the case of familiar compared to unfamiliar nests (Supplementary File 1, experiments QT1-QT2). These results indicate that the switch to transport is not strictly controlled by a fixed population threshold inside the new nest, but is also influenced by other sources of information. Additionally, we showed that the absence or presence of chemical cues inside the familiar nest had no significant effect on assessment time (Supplementary File 1, experiments P1-P2). We then investigated the role of individual memory. We compared nest evaluation by emigrating colonies (n = 16) that had been freely allowed to visit the new nest for one week prior to emigration (informed colonies moving to a familiar nest) or not (naïve colonies moving to an unfamiliar nest; Fig. 1). In informed colonies, workers that visited the new nest thus had the opportunity to memorise information about it before emigration, such as its quality or, more simply, its suitability as a housing site. Because we were specifically interested in testing the effect of memory, we suppressed all potential sources of social information inside the new nest during emigrations. Chemical cues were suppressed by removing the familiar nest just before initiating emigration, and replacing it with an identical nest that had never been visited by any ant. The environment outside the nest was left intact because in these ants chemical cues “en route to” or around the nest are not used by either informed or uninformed workers^30^. In addition, direct contacts were kept to a minimum by carefully controlling access to the new site during emigrations: only the first discoverer (the individual that first entered the new nest site after emigration was initiated) and workers that were then actively recruited to the new site were allowed in. First discoverers therefore had no access to social information provided by either chemical cues or direct contacts until they first recruited a nestmate to the new nest. Until the first recruitment, first discoverers thus only differed in their memory contents, which depended on whether they had previously visited the new nest or not.

**Figure 2:**
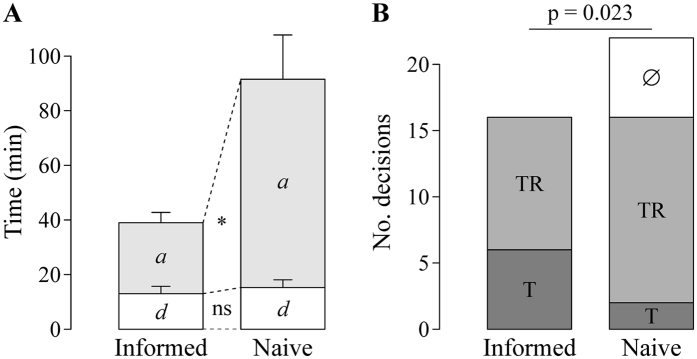
Results for memory experiment. (**A**) Discovery time (*d*) and assessment time (*a*) for informed and naïve colonies (n = 16). Bar lengths and whiskers represent means and standard errors, respectively. General linear mixed model (GLMM), effect of treatment, discovery time: χ^2^ = 1.31, df = 1, p = 0.25 (ns); assessment time: χ^2^ = 25.03, df = 1, p < 0.0001 (*). (**B**) First recruitment decisions by first discoverers in informed and naïve colonies (respectively; ∅: no recruitment; TR: tandem running; T: transport). The distribution of first recruitment decisions was compared between the two treatments using Fisher-Freeman-Halton’s exact test (p = 0.023). The sample size differs between the two treatments (informed: n = 16; naïve: n = 22) because when the first discoverer did not initiate recruitment within 90 min (∅), the new nest was replaced with another fresh nest, and the next worker that discovered it was considered first discoverer (see Methods for details).

Informed colonies assessed the new nest significantly faster than naïve colonies (Fig. 2A). This was associated with differences in the behaviour of first discoverers, which waited significantly less before initiating transport in informed than naïve colonies (40% lower transport latency; see Table 1 for statistical analyses). This could be explained by two non-mutually exclusive factors: (i) first discoverers in informed colonies spend less time navigating to and from the new nest because they have prior information about its location^30^,^31^; and (ii) first discoverers in informed colonies spend less time acquiring information inside the new nest before initiating transport. We found no evidence for the first hypothesis, as the inter-visit time (time spent outside the nest between successive visits) was similar between treatments (Table 1). However, three lines of evidence support the second hypothesis. First, first discoverers in informed colonies initiated transport after fewer visits, and those visits were significantly briefer. Overall, they spent roughly half the time inside the nest before initiating transport (Table 1). Second, first discoverers in informed colonies led fewer tandem runs and initiated transport at lower nest populations (Table 1), suggesting that they did not wait for a fixed quorum threshold to be met before initiating transport. Third, first discoverers differed significantly in their first recruitment decisions (Fig. 2B). All first discoverers in informed colonies initiated recruitment to the new site (n = 16), whereas some first discoverers in naïve colonies did not (n = 6). Additionally, when they did recruit, first discoverers in informed colonies were more likely to launch transport immediately, without leading any tandem runs (Fig. 2B). Transport, tandem running and no recruitment reflect respectively full, partial and no commitment to a nest^27^,^28^. Our results thus show that first discoverers were more strongly committed to the new nest in informed than in naïve colonies, even though there were no nest marking chemicals and no other workers inside the familiar nest when they first entered it. This shows that individual memory underlies the initial commitment of informed workers to the familiar nest.

**Figure 3:**
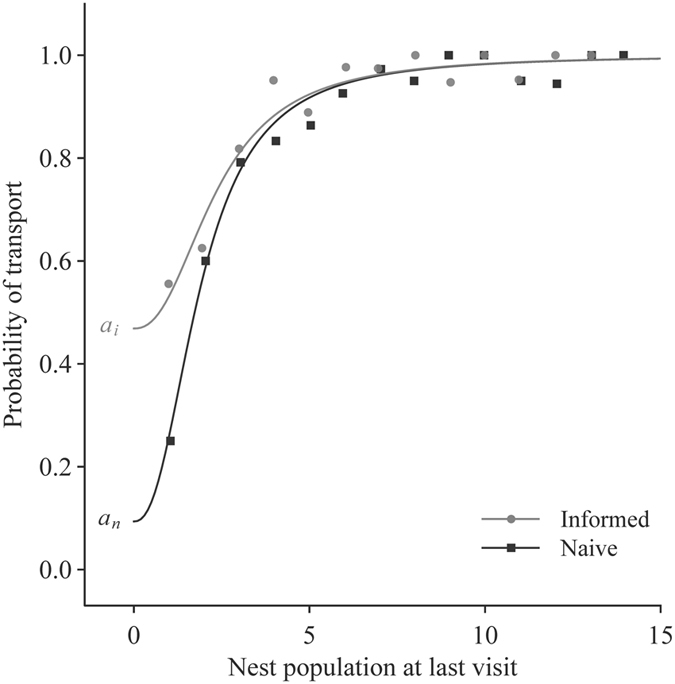
Quorum responses of informed and naïve colonies (memory experiment). Probability of recruiting workers deciding to initiate transport rather than tandem running, as a function of the nest population on their immediately preceding visit, for informed and naïve colonies (informed: n = 379; naïve: n = 427 recruitment decisions). Points represent the proportion of recruiters that initiated transport rather than tandem running for each nest population size. Lines represent the corresponding fitted quorum equations 
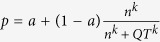
, where *p* represents the probability of carrying, *a* the probability of independent acceptance, *n* the nest population, *QT* the quorum threshold and *k* the steepness of the quorum response. All equations were fitted to the original raw data in which each recruitment decision was given a value of 0 (tandem run) or 1 (transport). Mean ± standard error of fitted parameters: *k = *2.36 ± 0.45; informed: *a*_*i*_ = 0.47 ± 0.072, *QT*_*i*_ = 2.3 ± 0.4; naïve: *a*_*n*_ = 0.094 ± 0.091, *QT*_*n*_ = 1.9 ± 0.2. Student’s t-tests with Benjamini–Hochberg correction, *QT*: t = 1.12, df = 780, p = 0.26; *a*: t = 3.24, df = 780, p < 0.005.

To investigate formally the quorum response of informed and naïve colonies, we fitted a non-linear ‘quorum equation’ inspired from a previous study of quorum responses^29^ to the recruitment decisions of all workers during emigrations (Fig. 3). We found that naïve and informed colonies did not differ in the value of their quorum threshold *QT*, but informed colonies showed a significantly higher probability of independent acceptance *a*, which represents the probability of initiating transport in the absence of any nestmates inside the nest. In other words, workers in informed colonies have a higher likelihood of fully committing to the new nest in the absence of social information, that is, using their private information only.

### Relationship between information certainty and relative use of private vs. social information

**Figure 4:**
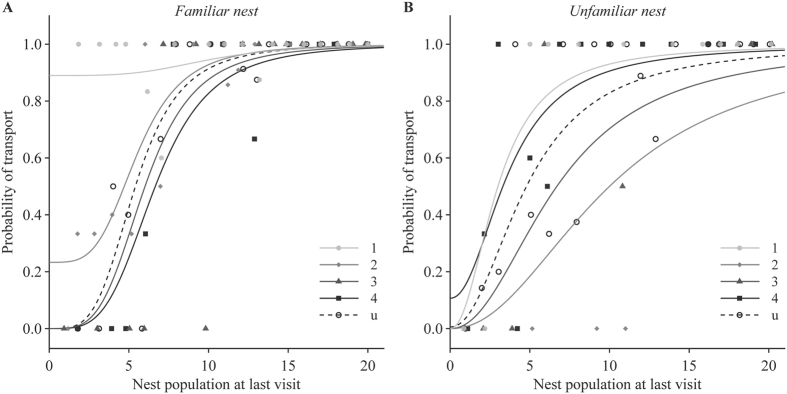
Quorum responses of informed workers depending on information certainty (nest-choice experiment). Analysis of data from Stroeymeyt *et al*.^30^, in which informed colonies had a choice between one familiar and one unfamiliar nest during emigrations. The points represent the proportion of recruiting workers deciding to initiate transport rather than tandem running, as a function of the nest population on their immediately preceding visit ((**A**): familiar nest; (**B**): unfamiliar nest). Data is shown separately for informed workers, which had visited the familiar nest during the exploration period, and for uninformed workers (*u*; ○), which had not. Informed workers were further classified as possessing either high (group 1), higher-intermediate (group 2), lower-intermediate (group 3), or low (group 4) information certainty depending on the number of visits they made to the familiar nest during exploration. The lines represent the corresponding fitted quorum equations. All equations were fitted to the original raw data in which each recruitment decision was given a value of 0 (tandem run) or 1 (transport). Mean ± standard error of fitted parameters: (**A**) *k = *3.8 ± 0.39; *a*_*1*_ = 0.89 ± 0.036, *QT*_*1*_ = 10 ± 2.9 (*n*_*1*_ = 266 recruitment decisions), *a*_*2*_ = 0.23 ± 0.06, *QT*_*2*_ = 5.5 ± 0.4 (*n*_*2*_ = 284), *a*_*3*_ = 0 ± 0.075, *QT*_*3*_ = 6.0 ± 0.3 (*n*_*3*_ = 142), *a*_*4*_ = 0 ± 0.065, *QT*_*4*_ = 6.6 ± 0.4 (*n*_*4*_ = 153), *a*_*u*_ = 0 ± 0.054, *QT*_*u*_ = 5.4 ± 0.2 (*n*_*u*_ = 474); (**B**) *k = *2.15 ± 0.2; *a*_*1*_ = 0 ± 0.08, *QT*_*1*_ = 3 ± 0.4 (*n*_*1*_ = 58), *a*_*2*_ = 0 ± 0.25, *QT*_*2*_ = 10 ± 2.8 (*n*_*2*_ = 35), *a*_*3*_ = 0 ± 0.09, *QT*_*3*_ = 6.7 ± 1.3 (*n*_*3*_ = 38), *a*_*4*_ = 0.1 ± 0.16, *QT*_*4*_ = 3.7 ± 0.9 (*n*_*4*_ = 78), *a*_*u*_ = 0.01 ± 0.047, *QT*_*u*_ = 4.8 ± 0.3 (*n*_*u*_ = 534).

The previous results indicate that informed workers acquired private information about the familiar nest prior to emigration, and thus were already partially committed to it at the onset of emigration. However, informed individuals may differ in the amount of information they acquired. To investigate this further, we analysed a dataset from a previous experiment which differed from the memory experiment in three ways: (i) all colonies were informed and had a choice between the familiar nest and an unfamiliar nest during emigrations; (ii) all pre-emigration visits to the familiar nest were recorded, and (iii) access to all nests was free throughout the experiment^30^. We found that informed workers that made the most visits to the familiar nest during exploration (group 1: top quartile, range 37–271, median 57 visits) had a significantly higher probability of independent acceptance *a* than any other group of informed workers (groups 2–4, see below) and than uninformed workers (Fig. 4A; Student’s t-tests with Benjamini–Hochberg correction: p < 0.0001 in all comparisons). Similarly, workers that made a higher-intermediate number of visits to the familiar nest (group 2: second highest quartile, range 17–36, median 24.5 visits) had a significantly higher probability of independent acceptance than the two groups of informed workers that made fewer visits to the familiar nest and than uninformed workers (p < 0.040 in all comparisons). The latter categories of informed workers (group 3: third quartile, range 7–16, median 11 visits; group 4: fourth quartile, range 1–6, median 3 visits) did not differ from one another or from uninformed workers in their probability of independent acceptance (p = 1 in all comparisons). There were no differences in the values of quorum threshold *QT* across categories (p > 0.15 in all comparisons).

These results are consistent with two hypotheses. First, high-exploration workers may initiate transport independently because of some intrinsic ‘personality’ characteristics; for example, there may be a general correlation between exploration activity and readiness to initiate transport. Under this hypothesis, we predict that workers informed about the *familiar* nest and later recruiting to the *unfamiliar* nest should display a similar correlation between number of visits and probability of independent acceptance. However, this was not the case: regardless of their previous exploration activity, all workers had a similarly low probability of accepting the unfamiliar nest independently (Fig. 4B; p > 0.88 in all comparisons). The relationship between number of visits and independent acceptance is therefore contingent on informed workers recruiting to the very nest they have information about.

A second hypothesis is that high-exploration workers increase their reliance on their private information because it is more reliable than that of low-exploration workers. Indeed, repeated visits to the familiar nest should reduce the uncertainty of private information by allowing workers to accumulate evidence about whether the nest is suitable through repeated information sampling^33^,^34^ and by facilitating memory formation^35^. Under this hypothesis, our results are consistent with a graded ‘copy-when-uncertain’ strategy: uninformed workers and the 50% informed workers with lower information certainty wait for social information to become available before committing to the new nest (*a*_*u*_ = *a*_*4*_ = *a*_*3*_ = 0), whereas well-informed workers are increasingly likely to commit to the new nest based on their private information only as the certainty of their private information is high (*a*_*2*_ = 0.23; *a*_*1*_ = 0.89). This hypothesis is supported by the results from the memory experiment, where the presence of privately-informed workers induced an increase in the probability of independent acceptance at the colony level (Fig. 3). In addition, pairwise comparisons of the behaviour of workers involved in the emigration in both treatments revealed clear changes in their recruitment behaviour (Table 2). These workers arrived at the new nest at similar stages of the emigration process in both treatments and therefore had access to similar levels of social information (Table 2). However, compared to naïve colonies, workers in informed colonies showed a significant reduction in (i) latency to first transport (57%), (ii) number of pre-transport visits (42%), (iii) total time spent inside the nest before transport (58%), (iv) nest population at which transport was initiated (30%) and (v) difference in nest population between first entrance and first transport (65%; Table 2). This provides further support for a causal relationship between private information and the tendency to not wait for social information to accumulate before initiating transport.

### Model simulations

Previous simulations of nest site selection by *Temnothorax* ants predicted that increasing the probability of independent acceptance *a* should result in faster, but less accurate and less cohesive decisions^29^. We modified the original simulation model to investigate how the graded increase in *a* highlighted in the previous paragraph affects colony performance (see Methods). We modelled a situation where colonies had to choose between two identical nest sites (*N*_*1*_ and *N*_*2*_). We assumed the presence of informed workers, of which a proportion *p*_*1*_ had previously visited *N*_*1*_ (the rest having visited *N*_*2*_). Informed workers were *w*_*d*_ times more likely to find the nest they knew about (familiar nest) than the other nest. In addition, informed workers were divided into three categories corresponding respectively to low, intermediate and high probabilities of independent acceptance of the familiar nest *a*, with an overall mean *a*_*i*_. Note that we did not make any assumptions about the quality of the information possessed by each category of informed workers. Instead, the model tests the effect of *a*_*i*_ on colony performance for a distribution of *a* among informed workers consistent with our empirical results.

**Figure 5:**
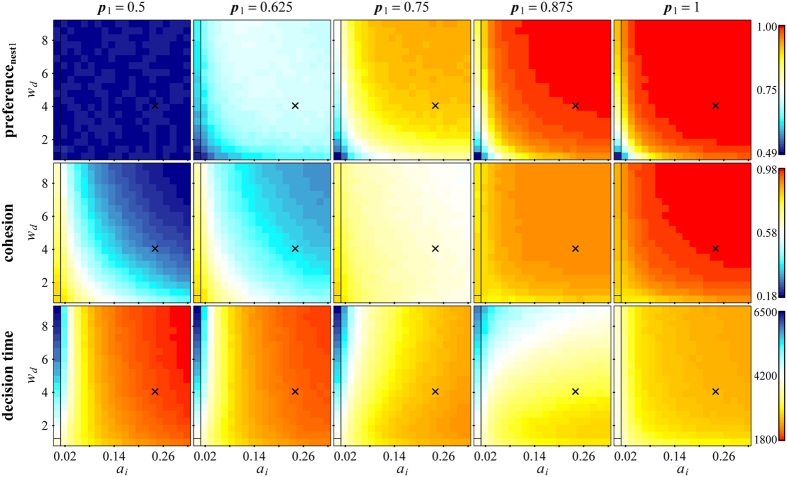
Model simulation results. Results from a stochastic model of emigrating colonies choosing between two identical nests, which they could visit prior to emigration. Results are shown for parameter values estimated from a previous empirical dataset^30^ (see Methods), but are qualitatively similar over a broad range of parameter values (Supplementary File 2). By definition, nest1 corresponds to the nest previously visited by a majority of informed workers. Simulations were run until all scouts were committed to a nest. Heatmaps show the outcome of emigrations, averaged over 10000 simulations for each combination of parameter values *a*_*i*_ (average probability of informed workers independently accepting the nest they know of) and *w*_*d*_ (relative likelihood of informed workers discovering the nest they know of rather than the other nest). The top row shows the preference for nest1, ranging from 0 (full preference for nest2) to 1 (full preference for nest1). The middle row shows colony cohesion, ranging from 0 (colonies equally split between both nests) to 1 (consensus for a single nest). The bottom row shows the decision time, expressed as simulation time steps. Results are shown for *p*_*1*_ (proportion of informed workers that visited nest1) ranging from 0.5 (left; as many informed workers for both nests) to 1 (right; choice between familiar nest1 and unfamiliar nest2). Within each panel, the left-most column corresponds to colonies using a fixed quorum rule, i.e. where all informed workers have the same low probability of independent acceptance as uninformed workers (*a*_*u*_); the framed bottom left corner corresponds to naïve colonies (*a*_*i*_ = *a*_*u*_ and *w*_*d*_ = 1), and the black cross corresponds to the values of *a*_*i*_ and *w*_*d*_ estimated from the empirical dataset^30^. Note that we assigned different values of *a* to different groups of informed workers according to the graded ‘copy-when-uncertain’ strategy, and that *a*_*i*_ represents the average of these values across all informed workers. Preference for the majority nest (nest1) and emigration speed (1/decision time) consistently increased as *a*_*i*_ increased. By contrast, an increase in *a*_*i*_ resulted in decreasing colony cohesion for *p*_*1*_ ≤ 0.75, but increasing colony cohesion for *p*_*1*_* = *0.875 (provided *w*_*d*_ is high enough) and for *p*_*1*_ = 1.

For all values of *p*_*1*_, decision speed and preference for the majority nest (when defined) increased with *a*_*i*_ (Fig. 5). The relationship between *a*_*i*_ and colony cohesion was more complex. If the asymmetry between the number of informed workers for *N*_*1*_ and *N*_*2*_ was low, colony cohesion decreased with *a*_*i*_ (Fig. 5, *p*_*1*_ ≤ 0.75). By contrast, if the asymmetry between the number of informed workers for *N*_*1*_ and *N*_*2*_ was high, colony cohesion increased with *a*_*i*_ (Fig. 5, *p*_*1*_ ≥ 0.875). All relationships were highly statistically significant (Supplementary File 3). In other words, providing that a sufficiently large majority of workers have private information about the same nest, informed colonies using the graded ‘copy-when-uncertain’ strategy are significantly faster, more cohesive and better able to follow the majority than informed colonies using a fixed quorum rule (i.e. where informed workers are as likely to accept the familiar nest independently as uninformed workers) and naïve colonies.

We performed additional simulations to investigate the performance of colonies using a simpler strategy, in which all informed workers have the same increased probability of independently accepting the familiar nest *a*_*i*_. These colonies showed qualitatively similar trends to colonies using the graded ‘copy-when-uncertain’ strategy, except that colony cohesion was found to increase with *a*_*i*_ only when all informed workers know about the same nest (*p*_*1*_ = 1; Supplementary File 3). Statistical analysis showed that colonies using the graded ‘copy-when-uncertain’ strategy are significantly more cohesive and better able to follow the majority, but slower than colonies where all informed workers behave in a homogeneous way (Supplementary File 3).

### Leadership

To get a better understanding of the influence of each worker category on the collective decision, we investigated the identity of pre-quorum recruiters, defined as the first workers recruiting to each nest until the quorum threshold is attained (Supplementary File 4). These workers are more influential than later workers, because once the quorum threshold is exceeded, subsequent workers automatically commit to the nest regardless of their private evaluation of that nest. Both simulation and experimental results revealed that informed workers with high probability of independent acceptance *a* were significantly more numerous among pre-quorum recruiters than expected given their proportion in the colony (Supplementary File 4). Informed workers with high *a* thus have the highest individual influence on the collective decision, indicating that they emerge as decision leaders through their early commitment to the familiar nest.

## Discussion

The temporal decoupling of information gathering and decision-making can provide substantial benefits^36^,^37^,^38^. In house-hunting ants, prior information gathering allows emigrating colonies to expedite the discovery and evaluation of familiar nests, thereby increasing emigration speed, accuracy and cohesion^31^. Our experimental results showed that the private memories of informed workers play a major role in increasing the pace of evaluation of familiar nests. First discoverers in informed colonies showed strong commitment to the familiar nest from the beginning of emigration, in the absence of any social information: as many as 37.5% launched transport before any other worker had entered the new nest. Additionally, the presence of privately-informed workers influenced the parameters of the quorum response of the whole colony: workers in naïve colonies waited for social information before switching to transport, whereas workers in informed colonies were more likely to make independent decisions solely based on their private information (higher probability of independent acceptance *a*).

To get a better understanding of the behaviour of informed workers, we used a previous dataset in which the exploration activity of each informed worker had been recorded individually^30^. We found that the 50% informed workers that made fewest visits to the familiar nest during exploration behaved like uninformed workers when evaluating that nest in later emigrations: they never made independent decisions (*a* = 0) and instead waited for social information before switching to transport. The 25% informed workers that made an intermediate-high number of visits to the familiar nest during exploration were more likely to start carrying without waiting for social information (*a* = 0.23), and the 25% that made most visits even more so (*a* = 0.89). These results are consistent with a graded ‘copy-when-uncertain’ social learning strategy: informed workers were all the more likely to switch to transport solely based on their private information as the certainty of their information was high (through memory reinforcement over repeated visits), and conversely, they were all the more likely to wait for social information before switching to transport as the certainty of their private information was low. This indicates that ants can use social information flexibly depending on the degree of certainty their private information, as do some vertebrates^2^,^3^,^4^,^32^,^39^,^40^. Previous experimental studies of social insects did not find this strategy in foraging ants^8^, though foraging honeybees use other social learning strategies (‘copy-when-costly’ and ‘copy-when-dissatisfied’)^1^,^24^,^41^,^42^. Collective foraging in social insects is a form of combined decision-making, i.e. it results from collective decisions that do not aim to reach consensus^11^. Though it is not observed in all ant species^43^, the prioritisation of private over social information is known to be beneficial in collective foraging, since it allows efficient allocation of workers over multiple food sources, discovery of new sources, and switching from sub-optimal to better sources^5^,^6^. Yet, it is more surprising in the context of consensus decision-making, since social information is crucial in ensuring that the group does reach consensus. Specifically in the case of nest site selection by ant colonies, theory predicts that a high probability of independent acceptance *a* should decrease both colony cohesion and decision accuracy in binary nest choices^29^.

We therefore performed simulations to investigate how the graded ‘copy-when-uncertain’ strategy might affect colony cohesion and other aspects of colony performance during emigrations involving a choice between two identical, good nests. Decision accuracy cannot be directly defined in this context because the two possible choices are identical. Instead, we considered the ability of colonies to make democratic decisions, that is, to choose the option supported by the majority of informed workers. Democracy and accuracy are usually aligned in collective decisions, because democratic decisions maximize the probability of making a correct choice when individual interests are aligned^10^,^14^, and maximise the group’s average fitness returns when individual interests are not aligned^44^,^45^. Our results on the ability of colonies to make a democratic decision can therefore be generalised to their ability to make an accurate choice in cases where available nest sites differ in quality. Our simulations predicted that colony cohesion, emigration speed and preference for the majority nest all increase with the average probability *a*_*i*_ of independent acceptance by informed workers, providing that a large enough majority of informed workers has information about the same nest (*p*_*1*_ ≥ 0.875 in Fig. 5). Under this condition, colonies using the graded ‘copy-when-uncertain’ strategy perform significantly better than naïve colonies and informed colonies using a fixed quorum rule in all three aspects. These predictions are in agreement with previous experiments showing that naïve colonies are slower, less cohesive and less accurate than informed colonies choosing between one familiar and one unfamiliar nests of identical quality (*p*_*1*_ = 1) or between two familiar nests of different quality (*p*_*1*_ = 0.93 ± 0.03 according to the number of workers observed inside or near each familiar nest just before emigration)^30^,^31^. By contrast, if informed workers are more evenly divided over both nests (*p*_*1*_ ≤ 0.75 in Fig. 5), increasing *a*_*i*_ decreases colony cohesion, and this effect is all the stronger as the asymmetry between nests is low. Why, then, do ants use a strategy that may favour colony splitting when there are multiple available nest sites? Previous studies showed that *Temnothorax albipennis* colonies put relatively less emphasis on cohesion than speed compared to closely related species^46^, and that split colonies are able to reunite shortly after emigration^31^. If reunification costs are low enough, it may be beneficial to use a strategy that enhances emigration speed and accuracy, even if it increases splitting risk in some conditions. Additionally, recent data suggests that splitting may have evolved as an adaptive strategy to reduce the time spent by the queen outside of a nest^47^. Alternatively, it may be that in natural conditions, cases where informed workers are divided over several nests are rare so splitting does not occur. Available nest sites in the field are unlikely to be exactly equivalent, and exploration for one week is long enough for a strong asymmetry to develop between nests of different quality (*p*_*1*_ = 0.93^31^). Even when confronted with a choice between four identical sites, colonies whose low-quality home nest was left intact are able to reach almost complete consensus within a few hours and move the large majority of the colony (mean ± standard error: 89.7 ± 3.7%) to a single new nest. This shows that symmetry-breaking deliberation occurs during exploration^48^. It is therefore likely that in natural conditions, deliberation mechanisms usually allow informed workers to reach complete or almost complete consensus over a single site before emigration is necessary. We thus expect the graded ‘copy-when-uncertain’ strategy to improve colony performance in terms of speed, accuracy and cohesion most of the time.

We argue that well-informed individuals emerge as decision leaders through the combined action of increased navigation efficiency^30^ and nest evaluation according to the graded ‘copy-when-uncertain’ strategy. This is supported by both our experimental and simulation results. Increasing either parameter *w*_*d*_ or *a*_*i*_ or the number of informed workers *n*_*i*_ consistently enhanced both preference for the majority nest and emigration speed (Fig. 5; Supplementary File 3). Importantly, this effect did not require explicit modelling of active communication such as chemical signalling or recruitment by tandem running. Instead, leadership by well-informed workers is an emergent property due to variation in individual reliance on private vs. social information. Well-informed workers indeed have a higher probability of independently accepting the familiar nest *a* and thus commit to the new nest earlier, as shown experimentally (Fig. 3). This in turn results in their disproportionate contribution to recruitment (Supplementary File 4) during the deliberation phase, which influences later decisions by less-well informed nestmates via quorum sensing. We have therefore identified a new simple behavioural rule at the individual level – increasing the relative reliance on private vs. social information as the quality of private information increases – that suffices to explain the emergence of individuals with high amount of private information as group leaders, without requiring active signalling or identification of these individuals as well-informed. This novel, simple rule could be of general importance for the emergence of well-informed leaders in self-organising animal and human groups. Leadership by knowledgeable individuals has indeed been repeatedly described in animals and humans (fish^49^,^50^,^51^; birds^52^,^53^; dolphins^54^; meerkats^55^; non-human primates^56^,^57^; humans^58^,^59^), but the precise behavioural rules that allow these individuals to consistently emerge as leaders, even when their identity is not known by other group members, are still poorly understood. Theoretical studies of collective movement in large groups previously predicted that knowledgeable individuals could emerge as leaders by being more ‘assertive’, i.e. by putting a relatively higher weight on personal preferences over social attraction^45^,^60^. Our study provides novel empirical support for this prediction. In addition, the strategy described here goes beyond the one modelled in previous theoretical work: it involves (i) variation in the degree of ‘assertiveness’ of informed workers, and (ii) a positive correlation between information certainty and degree of ‘assertiveness’. Our simulations show that introducing variation in the level of ‘assertiveness’ of informed workers suffices to allow colonies to make more democratic and more cohesive, albeit slower, choices than colonies where all informed workers behave in the same way (Supplementary File 3). Presumably, this is because variation in ‘assertiveness’ decreases the degree of conflict between alternative options. In addition, the graded ‘copy-when-uncertain’ strategy should further improve decision accuracy because it ensures that leadership is assumed by better-informed individuals, which should increase the likelihood that the better option is chosen^12^. We predict that these findings can be generalised to other types of collective decisions, and that the graded ‘copy-when-uncertain’ strategy may favour both accurate and cohesive decisions in other biological systems.

Finally, our results confirm quorum responses as a powerful decision-making mechanism, providing both robustness and flexibility^61^. Multiple studies have shown that animal groups can fine-tune the parameters of their quorum response, allowing them to adjust their decisions to current conditions adaptively without requiring the involvement of novel behaviours (ants^28^,^61^,^62^,^63^; fish^64^; humans^65^). Here we show that quorum response parameters can also be fine-tuned *within groups*, thereby granting individuals with different knowledge and experience appropriate weights in the decision process, which in turn improves the collective decision. This sheds new light on how animal groups can exploit individual variation in information quality to improve their decisions through the fine-tuning of individual behaviour, without requiring costly signalling or the identification of well-informed individuals. In addition, this highlights that measuring the quorum response as a single, homogeneous group response may therefore mask crucially important behavioural diversity within the group, and we encourage future studies of collective behaviour to investigate individual variation in greater detail.

## Methods

### Memory experiment

Twenty *T. albipennis* colonies were collected in Dorset, UK, in January 2010, and brought to Bristol, UK, where they were kept in artificial conditions^25^.

Throughout the experiment, we used artificial nests consisting of a cardboard perimeter sandwiched between two glass slides (50 × 76 mm), with an internal cavity of 35 × 50 mm, a ceiling height of 1.8 mm and an entrance tunnel of 2 × 8 mm. All nests were covered with an opaque cardboard sheet to make the interior dark. Previous work showed that *T. albipennis* colonies treat such nests as high-quality housing sites^25^,^30^,^31^.

Before the experiment, all workers in all colonies were individually marked with paint. Colonies were then positioned in the middle of the central dish and left to explore the arena for one week. At the end of exploration, the old nest was then destroyed and colonies had to move to a single high-quality new nest (NN in Fig. 1), identical to their old nest, positioned at one end of the arena. In control conditions, colonies had no available nest site to visit during exploration. They therefore emigrated to a single unfamiliar, novel nest site. In test conditions, colonies were allowed to familiarise themselves with the new nest during exploration. We then opened the familiar nest, and all workers inside were removed and released near the old nest. The familiar nest was then replaced with an identical, fresh, unmarked nest (i.e. a nest that had previously never been visited by any ant) and emigration was induced immediately thereafter. Chemical cues outside of the nest were not removed because a previous study showed that they do not influence the dynamics of discovery or evaluation of the new nest^30^. Analysis of data from that study further supports this claim: uninformed workers recruiting to either a familiar nest (previously visited by their nestmates) or to an unfamiliar nest (never visited by any worker) did not differ in the parameters of their quorum response (see below for details), showing that their evaluation of the new nest was unaffected by the potential presence of chemical cues outside the nest.

During emigrations, vertically sliding acetate doors fitted through the tunnels leading to the new nest site (Supplementary File 5) were used to control access of ants to the new site for both treatments. At the beginning of emigration, the sliding doors were open and allowed free passage to and from the peripheral dish containing the new nest. As soon as the new nest was entered by the first worker (‘first discoverer’), the doors were closed down and any other worker remaining in the peripheral dish was gently lifted with soft tweezers and released near the old nest. Sliding doors were then manually opened and closed so as to allow passage to the first discoverer, but to no other worker. After the first discoverer started to recruit nestmates to the new site (either by tandem running or by transport), passage was granted to the first discoverer and to all successively recruited ants, but to no other worker. Access control was carried out until a total of 20 workers had been recruited to the new nest, either by the first discoverer or by one of the successive recruits. If the first discoverer failed to recruit nestmates within 90 minutes of nest discovery, the new nest was replaced by an identical, fresh new nest. The next worker was allowed inside the new site and the experiment proceeded as explained above.

During emigrations, we recorded the discovery time (time interval between the destruction of the ON and the first ant entering the NN) and the assessment time (time interval between the first ant entering the NN and the first adult or brood item being actively carried by a nestmate into the NN). Webcams (Logitech ^®^ QuickCam ^®^ Communicate Deluxe) connected to motion detector software Webcam Zone Trigger Version 2.300 Pro (Omega Unfold. Inc.) were used to record all visits to the new nest by individually marked workers. Picture analysis later allowed us to determine the recruitment decisions (tandem running vs carrying) by all recruiters, and the nest population on the visit immediately preceding each recruitment decision. In addition, for each first discoverer (thereafter ‘focal worker’), we recorded (i) the number of tandem runs led and, if tandem running occurred, the time between the focal worker first entering the nest and first leading a tandem run (tandem running latency); (ii) the time between the focal worker first entering the nest and first carrying a nestmate or a brood item into the new nest (carrying latency); (iii) the total number of visits to the new nest before the focal worker’s first transport act (‘pre-carrying visits’); (iv) the duration of each pre-carrying visit and the time elapsing between successive pre-carrying visits (‘inter-visit time’); and (v) the maximum nest population during the visit that immediately preceded the focal worker’s first transport (individual quorum threshold).

Every colony experienced both control and test conditions in a pseudo-random order: half the colonies experienced control conditions first, and the other half experienced test conditions first. After experiencing one treatment, colonies were left undisturbed for at least one week before being tested in the second treatment to minimise the effects of previous experience. Colonies which had not discovered the familiar nest and colonies which had prematurely moved to the familiar nest during the exploration period were not included in the analysis, which resulted in a final sample size of 16 colonies.

It must be noted that although all first discoverers in the control were naïve, we cannot be sure that all first discoverers in the test were informed (i.e. had visited the new nest before emigration), since we did not record individual visits to the familiar nest during the exploration phase. However, a previous study using the same experimental design^30^ found that all first discoverers of the familiar nest were informed workers (n = 10 colonies), so we are confident that a high proportion (if not all) of first discoverers were also informed in the current experiment.

### Statistical analyses

All statistics were performed using R 3.0.2. The effect of treatment on the behaviour of first discoverers was analysed using general linear mixed models (GLMM) with ‘Treatment’ as a fixed effect and ‘Colony’, ‘Date of experiment’ as random effects; ‘Ant identity’ was added as an additional random factor for the analyses on pre-carrying visit duration and inter-visit time. GLMMs were implemented using R packages ‘lme4’ and ‘lmerTest’. Statistical significance was tested using a type III analysis of deviance with Wald chi-square test using R package ‘car’. Normality of residuals was checked using Shapiro-Wilk tests. When necessary, we transformed the data using either the logarithm or square root functions to ensure normality of residuals. Finally, first recruitment decisions among first discoverers were compared between treatments using Fisher-Freeman-Halton’s exact test.

### Formal study of the quorum response

Each recruitment decision was given a value of either 0 (tandem run) or 1 (transport). We then fitted the following quorum equation to the recruitment data for each treatment:


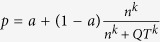
’ where *p* is the probability that a recruiter initiates transport rather than tandem running, *a* is the probability of independent acceptance (i.e. probability of transport in the absence of contacts with nestmates), *n* is the number of nestmates already present in the nest, *QT* is the quorum threshold, and *k* determines the steepness of the quorum response (adapted from ref. 29). Because preliminary analyses showed that there was no difference between treatments in the estimates for parameter *k*, we used a fixed value of parameter *k* (estimated by fitting the equation to the whole dataset). Parameter estimates for *a* and *QT* were compared across treatments using Student’s t-tests with Benjamini–Hochberg correction for multiple testing.

### The role of information certainty on the relative use of private vs. social information

In order to test whether information certainty influences the degree to which workers rely on private rather than social information, we used a dataset from a previous experiment^30^. In that experiment, the experimental design was identical to the one used in this study, except that (i) all colonies were informed and all visits to the familiar nests were monitored during the exploration period; (ii) at the time of the emigration, an identical, novel nest was added at the opposite end of the arena, so that colonies had a choice between a familiar and an unfamiliar nest; (iii) workers had free access to both nests during emigration^30^. Previous studies on learning showed that memory strength increases with repeated exposures to the learnt stimulus^35^. Accordingly, we determined four categories of informed workers (1: high, 2: higher-intermediate, 3: lower-intermediate and 4: low certainty of information) by dividing the total number of visits to the familiar nest before emigration started into quartiles (thus, the 25% workers that made most visits to the familiar nest during exploration were assigned to category 1, etc.). We then fitted quorum equations to the recruitment decisions for each category of informed workers and for uninformed workers at each nest, and we compared estimates for parameters *a* and *QT* between categories following the same procedure as described above. Crucially, uninformed workers showed no differences in their quorum response at the familiar or at the unfamiliar nest (*a*: t = 0.081, df = 983, p = 0.94; *QT*: t = 1.48, df = 983, p = 0.28), showing that their evaluation of the nest was not influenced by the presence of social chemical cues either outside or inside the familiar nest.

### Model simulations

We developed a stochastic simulation model inspired from a previous study by Sumpter and Pratt (2009) to simulate nest choice by emigrating ant colonies. Our model differed from the original model in that colonies had a choice between two identical rather than two different nests (thereafter named *N*_*1*_ and *N*_*2*_), and we explicitly modelled differences in private information among individuals. We assumed that emigrations were organised by *n* = 40 scouts (as in the original model), of which a number *n*_*i*_ were informed, i.e. already had private information about one of the two nests at the beginning of emigration. Among informed workers, a proportion *p*_*1*_ had information about *N*_*1*_, and (1-*p*_*1*_) about *N*_*2*._ We set *p*_*1*_ ≥ 0.5 so that the majority nest (when there was one) was always *N*_*1*_. We assumed that both nests were empty at the beginning of the simulation, and that workers discovered new nests according to a stochastic process with constant rate *d*. Uninformed workers were as likely to discover either site, whereas informed workers were *w*_*d*_ times more likely to discover the site they had information about than the other site. Explicitly modelling recruitment by committed scouts consistently increased both colony cohesion and emigration speed, but did not change the results qualitatively (Supplementary File 2). We therefore consider here that all nest discoveries were independent. Once a scout discovered a nest, she committed to it with a probability defined by the following quorum equation: 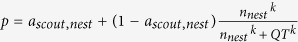, where *n*_*nest*_ is the nest population at the time of discovery (number of scouts already committed to the nest + discoverer), *QT* the quorum threshold, *k* the steepness of the quorum threshold, and *a*_*scout*,*nest*_ the probability of the considered scout independently accepting the considered nest. In agreement with our experimental results, *QT* and *k* were kept constant across nests. Uninformed workers were given the same, low probability of independently accepting either nest *a*_*u*_. Informed workers were also given probability *a*_*u*_ of independently accepting the nest they had no information about (unfamiliar nest). The probability of informed workers independently accepting the nest they did have information about (familiar nest) was defined as follows. For each nest, informed workers were divided into 3 categories at the beginning of the simulation: a proportion *p*_*low*_ of informed workers were assigned to the ‘low probability of independent acceptance *a*’ category, and the rest were equally divided into ‘intermediate *a*’ and ‘high *a*’ categories. The probability of independently accepting the familiar nest was *a*_*u*_ for ‘low *a’* workers, *a*_*f*_ for ‘intermediate *a*’ workers, σ.*a*_*f*_ for ‘high *a*’workers, with *a*_*f*_ ≥ *a*_*u*_ and σ ≥ 1. These resulted in an average probability of independent acceptance 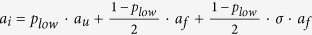 (1) for informed workers at the familiar nest. Commitment to a nest was considered irreversible, and the simulation was continued until all scouts were committed to a nest. At that time, we recorded (i) the number of time steps *T* required to complete the simulation; (ii) the cohesion of the colony: 
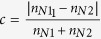
, where *n*_*N1*_ is the number of scouts committed to *N*_*1*_ and *n*_*N2*_ the number of scouts committed to *N*_*2*_; and (iii) whether there was a significant preference for either nest (tested using a binomial test on the number of scouts committed to *N*_*1*_ with p = 0.5), and if so, the identity of the preferred nest. For each combination of parameter values, we performed 10,000 simulations. We then calculated (i) the average simulation time, used as proxy for decision time; (ii) the average colony cohesion; and (iii) the relative preference for *N*_*1*_: 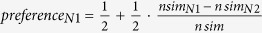, where *nsim* is the total number of simulations performed (i.e. 10,000) and *nsim*_*N1*_ (resp. *nsim*_*N2*_) the number of simulations in which a significant preference for *N*_*1*_ (resp. *N*_*2*_) was observed. Relative preference for *N*_*1*_ thus ranged from 0 (significant preference for *N*_*2*_ in all simulations) to 1 (significant preference for *N*_*1*_ in all simulations). Results are shown in the main text for the following parameter values, estimated from experimental results: *n*_*i*_ = 24 informed workers^30^, *QT* = 5.6 and *k* = 3.8 (estimated from recruitment decisions by all workers at the familiar nest, see Fig. 4), *a*_*u*_ = 0.01, *p*_*low*_ = 0.5 (categories 3 and 4 of informed workers, represent the 50% of informed workers with lower information certainty, have an equally low probability of independent acceptance, see Fig. 4) and σ = 4 (*a*_*1*_ ≈ 4.*a*_*2*_, see Fig. 4). Results for different parameter values are presented in Supplementary File 2. We systematically varied *w*_*d*_ between 1 and 9 and *a*_*f*_ between *a*_*u*_ and 1/σ (so that σ.*a*_*f*_ ≤ 1); for each value of *a*_*f*_, *a*_*i*_ was calculated according to formula (1). Note that the special case of naïve colonies (i.e. no informed workers) corresponds to *w*_*d*_ = 1 and *a*_*i*_ = *a*_*u*_ (equal likelihood of discovering and of independently accepting either nest for all worker). The special case of the ‘copy-when-informed’ strategy was obtained by setting *p*_*low*_ = 0 and σ = 1 (all informed workers having the same probability of independently accepting the familiar nest).

## Additional Information

**How to cite this article**: Stroeymeyt, N. *et al*. Information Certainty Determines Social and Private Information Use in Ants. *Sci. Rep.* **7**, 43607; doi: 10.1038/srep43607 (2017).

**Publisher's note:** Springer Nature remains neutral with regard to jurisdictional claims in published maps and institutional affiliations.

## Supplementary Material

Supplementary Information

Supplementary Dataset 1

Supplementary Dataset 2

Supplementary Dataset 3

Supplementary Dataset 4

Supplementary Dataset 5

## Figures and Tables

**Table 1:** Statistical analyses for first discoverers (memory experiment).

Variable	Treatment	Value	Test statistic	P-value
Transport latency	Informed	27.7 ± 4.3 (min)	χ^2^ = 7.57	*p* = 0.006
	Naïve	47.7 ± 8.7 (min)		
No. pre-transport visits	Informed	4.8 ± 0.5	χ^2^ = 75.67	*p* < 0.0001
	Naïve	6.3 ± 0.7		
Pre-transport inter-visit time	Informed	5.2 ± 0.5 (min)	χ^2^ = 1.20	*p* = 0.27
	Naïve	6.2 ± 0.5 (min)		
Pre-transport visit duration	Informed	1.3 ± 0.1 (min)	χ^2^ = 4.23	*p* = 0.04
	Naïve	1.9 ± 0.2 (min)		
Cumulated time spent in nest before transport g (min)	Informed	6.3 ± 1 (min)	χ^2^ = 6.93	*p* = 0.0085
	Naïve	11.7 ± 2.7 (min)		
No. tandem runs led	Informed	2.1 ± 0.6	χ^2^ = 14.11	*p* < 0.0005
	Naïve	2.6 ± 0.5		
Nest population at first transport	Informed	2.2 ± 0.4	χ^2^ = 12.46	*p* < 0.0005
	Naïve	4.1 ± 1		

Test statistics (Wald χ^2^) and p-values are provided for the effect of treatment on each variable (GLMM with colony and date of experiment as random factors).

**Table 2:** Statistical analyses for individuals actively involved in the emigration in both treatments (memory experiment).

Variable	Treatment	Value	Test statistic	P-value
Entrance rank	Informed	5.6 ± 1	V = 26	*p* = 0.29
	Naïve	5 ± 1.3		
Entrance time	Informed	47.6 ± 9.8 (min)	V = 31	*p* = 0.90
	Naïve	47.4 ± 9.7 (min)		
Nest population at first entrance	Informed	3.9 ± 1	V = 26.5	*p* = 0.26
	Naïve	3.3 ± 0.9		
Transport latency	Informed	16.5 ± 3.7 (min)	V = 5	***p* < 0.01**
	Naïve	39 ± 9.2 (min)		
No. pre-transport visits	Informed	2.7 ± 0.4	V = 0	***p* = 0.022**
	Naïve	4.7 ± 0.8		
Cumulated time spent in nest before transport (min)	Informed	6.5 ± 1.3 (min)	V = 10	***p* = 0.042**
	Naïve	15.3 ± 4.7 (min)		
Nest population at first transport	Informed	5.5 ± 1	V = 2.5	***p* = 0.020**
	Naïve	7.9 ± 0.9		
Increase in nest population between first entrance and first transport	Informed	1.6 ± 0.7	V = 2.5	***p* = 0.012**
	Naïve	4.6 ± 1.2		

Test statistics and p-values are provided for the effect of treatment on each variable (Wilcoxon matched-pairs tests; n = 11).
