# Supplementary material for: Information Certainty Determines Social and Private Information Use in Ants
Source: Sci Rep. 2017 Mar 3;7:43607. doi: 10.1038/srep43607 (PMC5356460; doi:10.1038/srep43607)
Supplement: Supplementary Information [file srep43607-s1.pdf]

Information certainty determines social and private information use in ants

Stroeymeyt N., Giurfa M., Franks N. R.

Supplementary File 1
Preliminary experiments: role of direct contacts and chemical cues in expediting familiar nest evaluation

Four experiments were performed to investigate the role of two forms of social information in expediting familiar nest evaluation: direct contacts and chemical cues (see Methods).

Results

1. The role of direct contacts

Nest population should grow faster in familiar than unfamiliar nests because (i) there are already some workers in the familiar nest at the onset of emigration and (ii) the discovery rate of the familiar nest is greater, giving it a head start in the recruitment process[1,2]. The faster assessment of familiar nests could thus be a direct consequence of these nests attaining the quorum threshold earlier than unfamiliar nests. If this mechanism alone accounts for expedited familiar nest evaluation, then we expect that (i) decisions to switch to transport should occur at similar nest populations for all nests, and (ii) delaying nest population growth, for example by clearing the familiar nest of workers at the onset of emigration, should delay decisions to switch to transport. Experiments QT1 and QT2 aimed at testing these predictions (Table 1).

	Treatment	Private information	Chemical marking	Direct contacts: Workers in	Direct contacts: Free access
**QT1**	Control	no	no	no	yes
n=19	Test	yes	yes	no	yes
**QT2**	Control	yes	yes	yes	yes
n=18	Test	yes	yes	no	yes
**P1**	Control	yes	yes	no	yes
n=18	Test	yes	no	no	yes
**P2**	Control	no	no	no	yes
n=22	Test	no	yes	no	yes

Table 1. Experimental designs. For each experiment, sources of information that were available ('yes') and non-available ('no') during emigration are listed. Direct contacts in the new nest depended on whether there were workers inside the new nest at the onset of emigration ('Workers in'), and whether we allowed all workers free access to the new nest or whether we limited access to the new nest to specific workers. Informed workers that were removed from familiar nests at the onset of emigration were returned to their old nest.

In experiment QT1, colonies moving to a familiar nest that was cleared of workers at the onset of emigration were significantly faster than colonies moving to an unfamiliar nest (Figure S1 A). This was at least in part due to evaluation of familiar nests being expedited compared to unfamiliar nests: assessment time was significantly shorter, fewer tandem runs were led, and workers made fewer and shorter visits to familiar than unfamiliar nests before transport started (Table 2). As predicted, nest population increased faster in familiar than in unfamiliar nests, which could contribute to familiar nests attaining the quorum threshold earlier. However, contrary to our expectation, scouts switched to transport at significantly lower nest populations for familiar than unfamiliar nests, that is, they used significantly lower quorum thresholds for familiar than unfamiliar nests (Figure S1A; Table2).

	Emig. time	Assess. Time	No. TR	TR latency	No. visits	Visit duration	QT	Time to QT	Carry. latency	Pop. increase
**QT1** *n=19*	↘ χ^2=26.25 ***p*<1.10^{-6}**	↘ χ^2=38.47 ***p*<1.10^{-9}**	↘ χ^2=11.51 ***p*<1.10^{-3}**	→ χ^2=2.19 *p*=0.14	↘ χ^2=8.11 ***p*<0.01**	↘ χ^2=20.45 ***p*<1.10^{-5}**	↘ χ^2=6.08 ***p*=0.014**	↘ χ^2=36.22 ***p*<1.10^{-8}**	-	↗ χ^2=24 ***p*<1.10^{-6}**
**QT2** *n=18*	→ χ^2=1.34 *p*=0.25	→ χ^2=0.13 *p* = 0.71	→ V=4 *p*=0.85	→ V=0 *p*=0.5	→ χ^2=0.21 *p*=0.65	↗ χ^2=6.72 ***p*<0.01**	↘ χ^2= 4.83 ***p*=0.028**	→ χ^2=0.037 *p*=0.85	-	-
**P1** *n=18*	→ χ^2=1.25 *p*=0.26	↗ χ^2=3.69 *p*=0.055	↗ χ^2=6.65 ***p*<0.01**	→ χ^2=0.95 *p*=0.33	→ χ^2=0.053 *p*=0.82	→ χ^2=1.36 *p*=0.24	→ χ^2=0.99 *p*=0.32	↗ χ^2=4.83 ***p*=0.028**	-	↘ χ^2=17.71 ***p*<1.10^{-3}**
**P2** *n=22*	↘ χ^2=5.36 ***p*=0.02**	↘ χ^2=3.64 *p*=0.056	→ V=65 p=0.79	↘ χ^2=4.87 ***p*=0.027**	→ V=130.5 p=0.91	→ χ^2=0.098 *p*=0.75	→ χ^2=1.24 p=0.27	→ χ^2=1.16 p=0.28	-	↗ χ^2=8.84 ***p*<0.01**

Table 2. Statistical analyses.
Arrows indicate that the focal variable was significantly higher (↗) or lower (↘) in test than in control conditions, or similar (→) between the two treatments. For each analysis, the test statistic (Wald χ^2 for GLMM and V for Wilcoxon matched-pairs tests) and p-value are given for the effect of the interaction between time and treatment (population increase) or treatment only (all other variables).

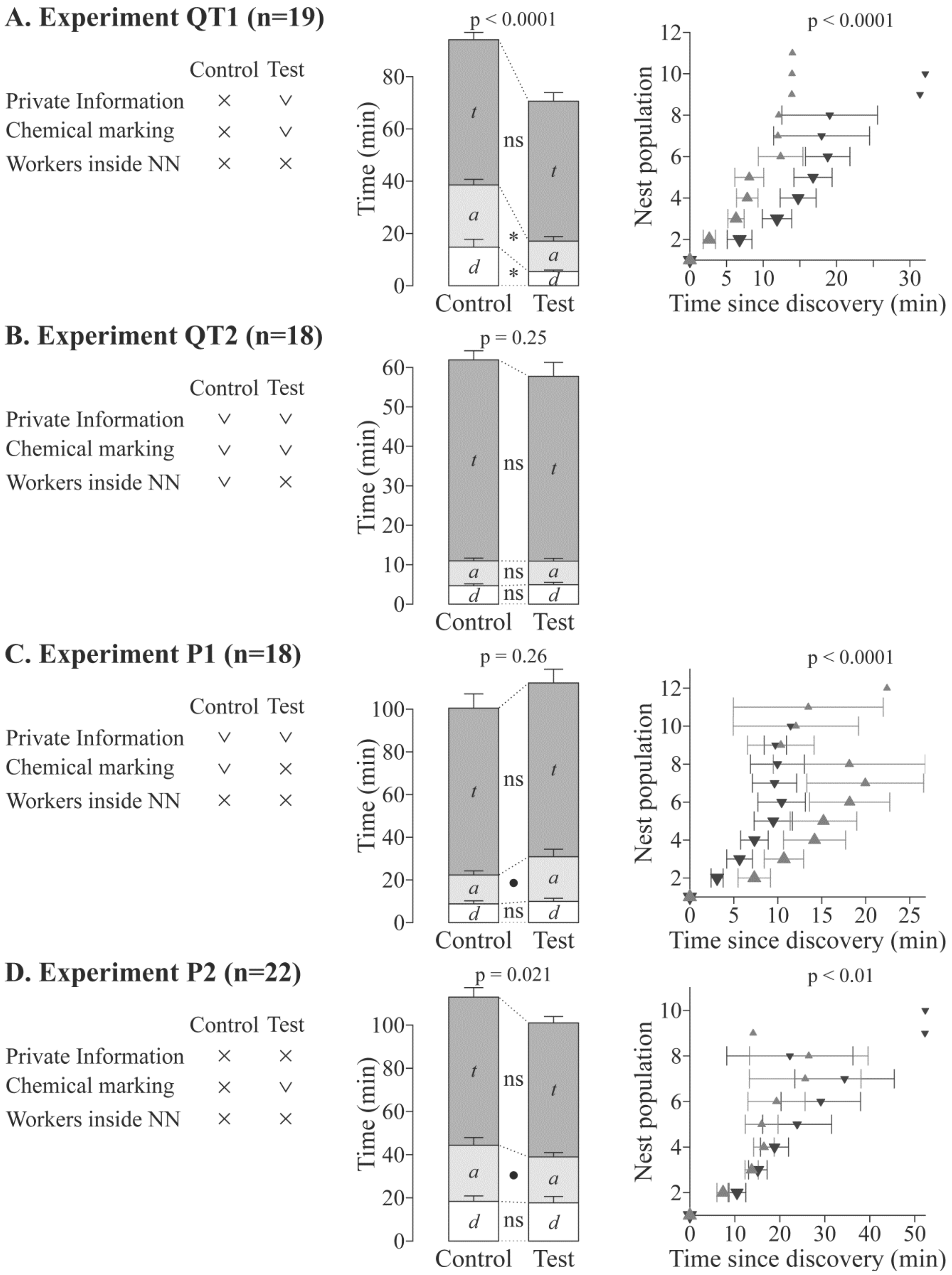

Figure S1. Results for all experiments. (A-D) Left: experimental design, showing for each treatment which sources of information were available at the beginning of the emigration (∨) and which were not (×). Middle: emigration time and each of its components (*d*: discovery, white; *a*: assessment, light grey; *t*: transport, dark grey) for test (top) and control colonies (bottom). Bar lengths and whiskers represent means and standard errors, respectively. P-values are given for the effect of treatment on emigration time (top) and each of its components (ns: $p>0.06$; •: $0.05<p\leq0.06$; *: $p\leq0.05$). Right: nest population as a function of time since nest discovery for control (dark grey) and test colonies (light grey). Triangles and whiskers represent the means and standard errors of the time to attain each nest population value. P-values are given for the effect of the interaction between time and treatment on nest population.

In experiment QT2, contrary to our expectation, clearing familiar nests of workers at the onset of emigration (test) did not slow down nest assessment or nest emigration relative to familiar nests that were not cleared of workers (control). There were no differences between treatments in the number of visits to the new nest before transport started, in tandem running behaviour, or in the time to attain the quorum threshold. Importantly, carrying was initiated at significantly lower nest populations in test than in control colonies, probably due to the initial depletion of workers inside the new nest in the test (Figure S1B; Table 2).

Overall, these two experiments do not support the hypothesis that faster nest population growth alone accounts for the faster assessment of familiar nest sites. Indeed quorum threshold values differed for familiar and unfamiliar nests, and also differed for identical familiar nests depending on initial population conditions. Nest assessment time therefore did not solely reflect the time necessary for the nest population to attain a given, fixed quorum threshold. Other factors, such as chemical marking of the familiar nest or use of existing private information by informed workers, may also underlie the faster evaluation of familiar nests.

2. The role of chemical communication

Experiments P1 and P2 aimed at investigating the effect of chemical cues on familiar nest evaluation by informed (experiment P1) and naïve colonies (experiment P2; see Table 1).

Colonies moving to familiar nests that were cleared of workers at the onset of emigration showed no differences in overall emigration time whether chemical marking of the nest had been left intact (control) or removed (test; experiment P1). Decisions to start carrying were made after a similar number of visits and at similar quorum thresholds in test and control conditions, and workers did not change the duration of their visits to the new nest in reaction to the absence of chemical cues. However, workers led more tandem runs to the unmarked than to the marked nest. In addition, nest populations increased more slowly when chemical marking was removed from the familiar nest, presumably because workers engaged in the slow recruitment method of tandem running spent more time outside the nest. Accordingly, the quorum threshold was attained more slowly. However, the

duration of the assessment phase tended to be longer when chemical marks had been removed, although this trend was not statistically significant (Figure S1C; Table 2).

Naïve half-colonies moving to unfamiliar nests that had been previously visited by sister half-colonies emigrated significantly faster than naïve half-colonies moving to fresh, previously unvisited unfamiliar nests (experiment P2). Although test and control colonies did not differ in the number of tandem runs they produced, or in the number or duration of visits to the new nest before transport started, colonies that had access to chemical cues (test) initiated tandem running earlier and showed a faster increase in nest population. Since decisions to start carrying were made for similar quorum thresholds in the control and in the test, this resulted in the assessment phase tending to be shorter in test than in control conditions, although this trend was not statistically significant (Figure S1D; Table 2).

These two experiments suggest that chemical marking of the familiar nest by nestmates influences nest evaluation by both informed (P1) and naïve (P2) colonies in emergency emigrations, though this effect is limited and did not result in significantly faster nest evaluation.

Materials and Methods

General experimental design

Throughout experiments, colonies were housed in artificial nests consisting of a cardboard perimeter sandwiched between two glass slides (50×76 mm), with an internal cavity of 35×50 mm, a ceiling height of 1.8 mm and an entrance tunnel of 2×8 mm. All nests were covered with an opaque cardboard sheet to make the interior dark. Previous work showed that *T. albipennis* colonies consider such nests as high-quality housing sites[1-3].

All experiments were carried out in experimental arenas consisting of five interconnected Petri dishes with Fluon-coated walls, similar to those used in previous studies (Fig 1A;[1,2]). Colonies housed in high-quality nests (‘old nest’, ON in Fig 1A) were positioned in the middle of the central dish and left to explore the arena for one week. The old nest was then destroyed and colonies had to

move to a single high-quality new nest (NN in Fig 1A), identical to their old nest, positioned at one end of the arena.

During emigrations, we recorded the emigration time (time interval between the destruction of the ON and the last brood item being carried into the NN) and its three components: discovery time (time interval between the destruction of the ON and the first ant entering the NN), assessment time (time interval between the first ant entering the NN and the first adult or brood item being actively carried by a nestmate into the NN), and transport time (time interval between the first transport and the last brood item being carried into the NN). Additionally, we recorded the time between the first ant entering the nest and the first tandem run (tandem running latency) and the total number of tandem runs led from the old nest to the new nest during the assessment phase. Webcams (Logitech ® QuickCam ® Communicate Deluxe) connected to motion detector software Webcam Zone Trigger Version 2.300 Pro (Omega Unfold. Inc.) were used to record all visits to the new nest. This allowed us to determine the total number of visits to the new nest before transport started, as well as an approximate average visit duration (calculated as the cumulative time spent by all visitors inside the new nest divided by the number of visits before transport started). Logging successive entrances into and exits from the new nest also allowed continuous monitoring of the nest population over time. From this we determined an approximate quorum threshold for each emigration (maximum population attained in the new nest before the first transport). Despite fluctuations, the population in the new nest progressively increased over time between discovery and first transport, thus repeatedly reaching new, previously unattained maximal values. We recorded the times at which these successive new maxima were attained to evaluate the rate of nest population increase prior to transport.

In all experiments, every colony experienced both control and test conditions (detailed below) in a pseudo-random order: half the colonies experienced control conditions first, and the other half experienced test conditions first. After experiencing one treatment, colonies were left undisturbed for at least one week before being tested in the second treatment to minimise the effects of previous experience[4]. Colonies which had not discovered the familiar nest and colonies which had prematurely

moved to the familiar nest during the exploration period were not included in the experiment. Table 1 shows the final sample sizes for each experiment.

The role of direct contacts: experiments QT1 and QT2

Experiment QT1

In control conditions, colonies had no available nest to visit during exploration. They then emigrated to an unfamiliar new nest. In the test, colonies were allowed to familiarise themselves with the new nest during exploration. The familiar nest was then opened by lifting the top glass slide, and all workers present inside the nest were gently removed with soft tweezers and released near the old nest. The familiar nest was then closed back and emigration was induced immediately thereafter (Table 1).

Experiment QT2

In both control and test conditions, colonies were allowed to familiarise themselves with the new nest during exploration. In the test, the familiar nest was then opened and all workers inside were removed and released near the old nest. In the control, the familiar nest was also opened by lifting the top glass slide to induce similar disturbance, but workers were left inside the nest. In both treatments, the familiar nest was then closed back and emigration was induced immediately thereafter (Table 1).

The role of chemical communication: experiments P1 and P2

Experiment P1

In both control and test conditions, colonies were allowed to familiarise themselves with the new nest during exploration. The familiar nest was then opened, and all workers inside were removed and released near the old nest. In the control, the familiar nest was then closed (chemical cues present), whereas in the test, it was replaced with an identical, novel nest (chemical cues absent). Emigration was induced immediately thereafter (Table 1).

Experiment P2

Twenty-two colonies were each split into two equal halves. During exploration, ‘informed’ half-colonies were allowed to familiarise themselves with the new nest whereas ‘naïve’ half-colonies had no available nest to visit. In the control, naïve half-colonies were then induced to emigrate to an unfamiliar new nest. In the test, familiar nests from informed half-colonies were opened and all workers inside were removed. The nests were then closed and transferred to the arena of naïve half-colonies. Naïve half-colonies were then immediately induced to emigrate to the transferred new nest (Table 1). To avoid any confounding effects of nestmate recognition[5], transfers were always done between half-colonies from the same mother colony, as they usually maintain the same colony odour for several weeks in *Temnothorax* sp.[6].

References

1 Stroeymeyt, N., Franks, N. R. & Giurfa, M. Knowledgeable individuals lead collective decisions in ants. *J. Exp. Biol.* **214**, 3046-3054 (2011).

2 Stroeymeyt, N., Giurfa, M. & Franks, N. R. Improving decision speed, accuracy and group cohesion through early information gathering in house-hunting ants. *PLoS One* **5**, e13059, doi:10.1371/journal.pone.0013059 (2010).

3 Franks, N. R., Mallon, E. B., Bray, H. E., Hamilton, M. J. & Mischler, T. C. Strategies for choosing between alternatives with different attributes: exemplified by house-hunting ants. *Anim. Behav.* **65**, 215-223 (2003).

4 Langridge, E. A., Franks, N. R. & Sendova-Franks, A. B. Improvement in collective performance with experience in ants. *Behav. Ecol. Sociobiol.* **56**, 523-529 (2004).

5 Franks, N. R. *et al.* Avoidance of conspecific colonies during nest choice by ants. *Anim. Behav.* **73**, 525-534 (2007).

6 Stroeymeyt, N., Brunner, E. & Heinze, J. "Selfish worker policing" controls reproduction in a *Temnothorax* ant. *Behav. Ecol. Sociobiol.* **61**, 1449-1457, doi:10.1007/s00265-007-0377-3 (2007).

Supplementary File 2
Effect of model parameters on simulation outcome

The following three figures present the average simulation outcome for different values of parameters:

- n_i. number of informed workers (Figure S2)
- p_{low}, proportion of informed workers with low probability of independent acceptance (Figure S3)
- σ, commitment bias: $\sigma = \frac{a_{high\ a}}{a_{intermediate\ a}}$ (Figure S4)

In each figure, each panel shows simulation results (averaged over 10,000 simulations) for a given value of the focal parameter, the other parameters having the same values as in the main manuscript (n_i=24, p_{low}=0.5, σ=4). Data representation within each panel is similar to main manuscript Figure 5.

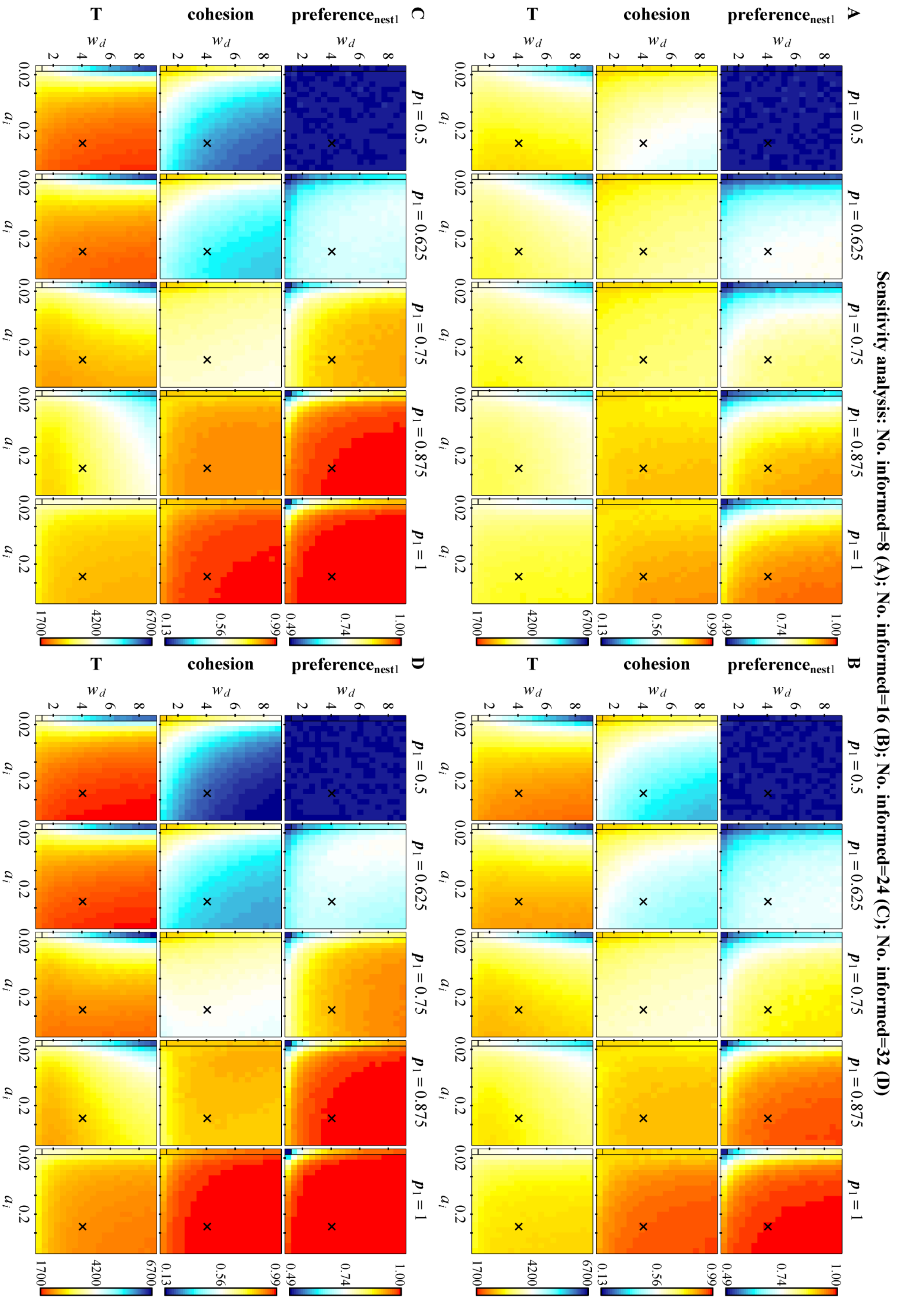

Figure S2. Effect of model parameters on simulation outcome: No. informed

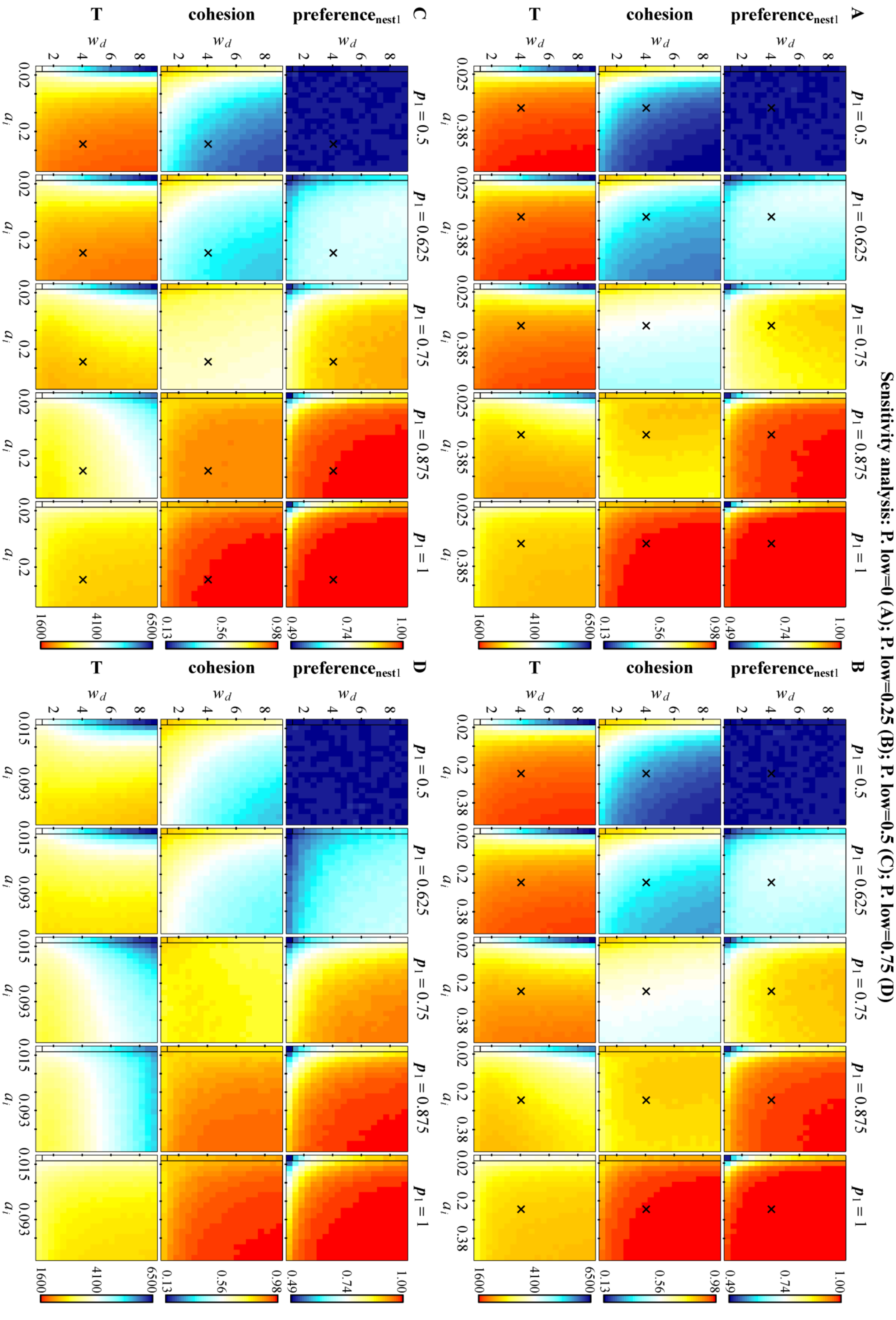

Figure S3. Effect of model parameters on simulation outcome: proportion of individuals with low *a*

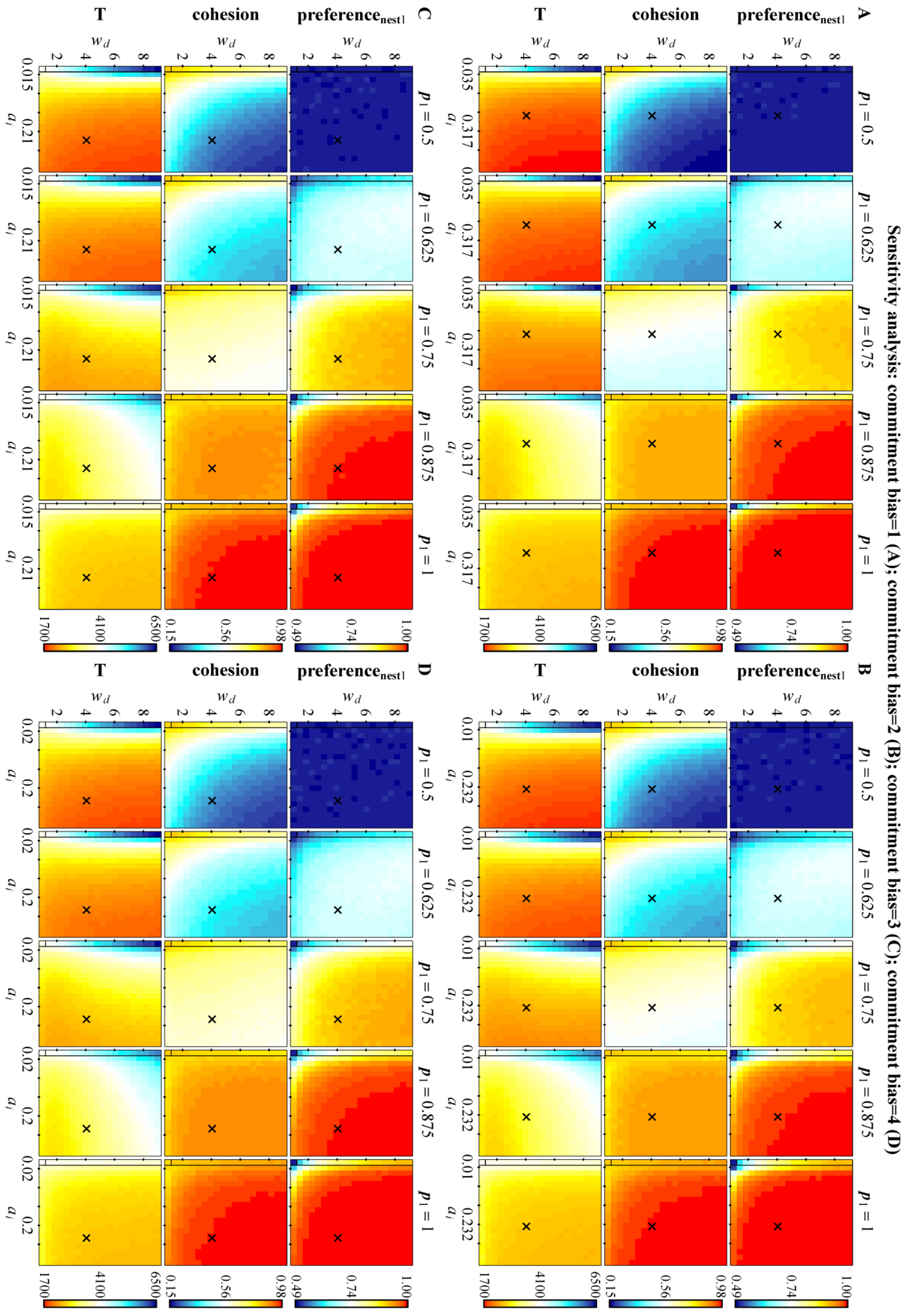

Figure S4. Effect of model parameters on simulation outcome: commitment bias

We also investigated the effect of recruitment on our simulation results. We defined d_{nest} the probability of workers discovering the focal nest (nest 1 if worker is uninformed; familiar nest if worker is informed) as follows:

$$\begin{cases} d_{nest} = p_{uncommitted} \cdot 0.5 + p_{committed} \cdot \frac{r_{nest}}{1+r_{nest}} & if\ worker\ is\ uninformed \\ d_{nest} = p_{uncommitted} \cdot \frac{w_d}{1+w_d} + p_{committed} \cdot \frac{w_d \cdot r_{nest}}{1+w_d \cdot r_{nest}} & if\ worker\ is\ informed \end{cases},$$

where $p_{uncommitted}$ is the proportion of uncommitted workers; $p_{committed}=1-p_{uncommitted}$; w_d is the relative likelihood of informed workers discovering the familiar nest relative to the unfamiliar nest, and r_{nest} is defined as follows:

$$\begin{cases} r_{nest} = 1 & if\ n_{nest} = n_{other\ \ nest} \\ r_{nest} = 1 + r \cdot \frac{n_{nest}}{n_{nest} + n_{other\ \ nest}} & if\ n_{nest} > n_{other\ \ nest} \\ r_{nest} = \frac{1}{1 + r.\frac{n_{other\ \ nest}}{n_{nest} + n_{other\ \ nest}}} & if\ n_{nest} < n_{other\ \ nest} \end{cases},$$

where n_{nest} is the number of workers already committed to the focal nest, $n_{other\ nest}$ the number of workers already committed to the non-focal nest, and r a parameter describing the recruitment advantage to the majority nest.

The following figure (Figure S5) presents the effect of parameter r on simulation outcome (note that r=0 corresponds to the situation where there is no recruitment, as in the main manuscript).

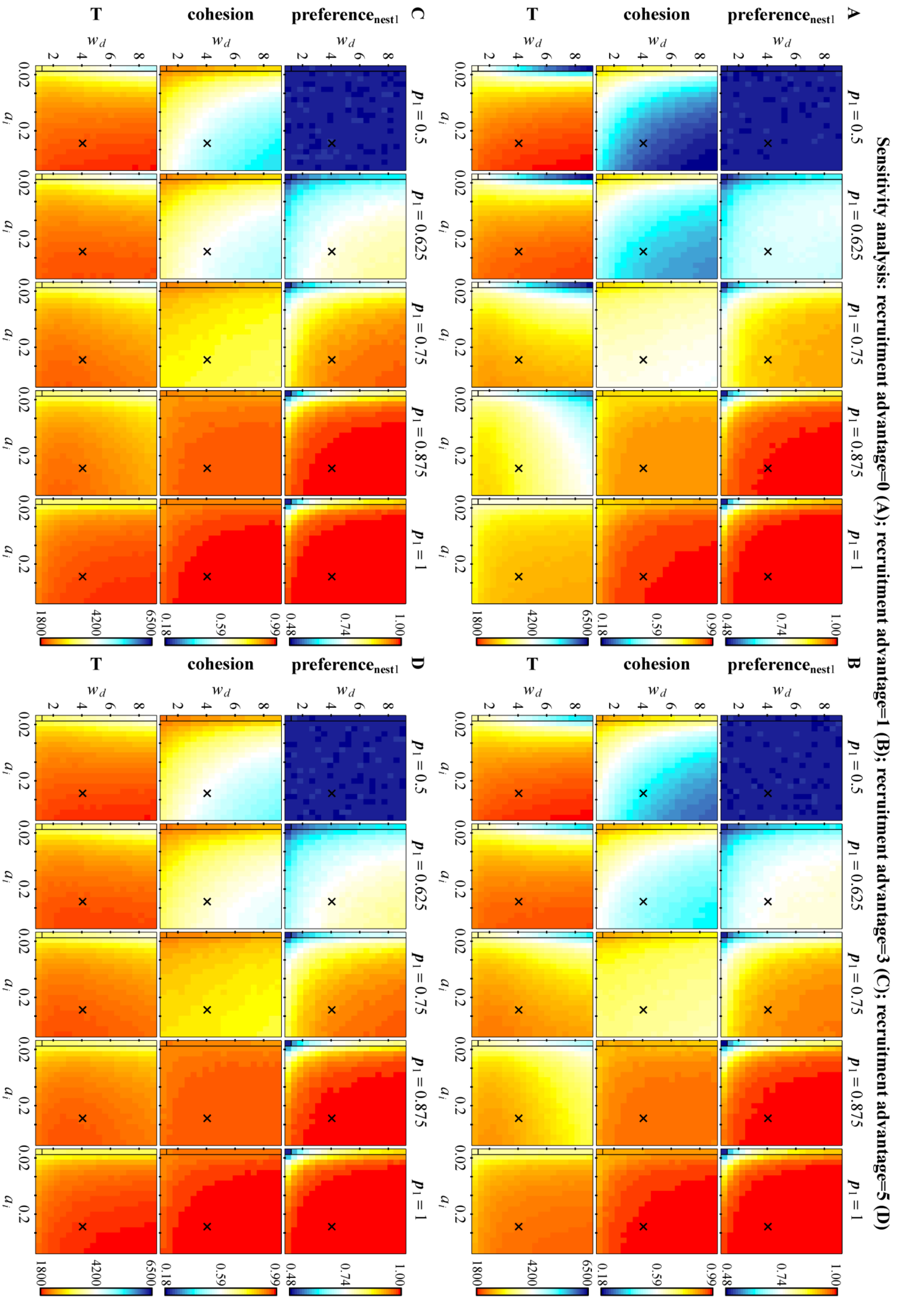

Figure S5. Effect of model parameters on simulation outcome: recruitment advantage

Supplementary File 3
Comparison of 'copy-when-uncertain' strategy with other strategies

I. Graded 'copy-when-uncertain' vs. fixed quorum rule

In order to facilitate data interpretation, we plotted the relative performance of colonies using the graded 'copy-when-uncertain' strategy compared to informed colonies using a fixed quorum rule (a_i=a_u and w_d≥1; Figure S6).

We used GLMM to test whether the accuracy, cohesion and emigration time of colonies using the graded 'copy-when-uncertain' strategy differed from the average performance of informed colonies using a fixed quorum rule. We found that overall, colonies using the 'graded copy-when-uncertain' strategies were faster ($F_{1,1997}$=754.69, p<1.10^{-15}), showed stronger preference for the majority nest ($F_{1,1997}$=50.71, p< 1.10^{-11}), but were less cohesive ($F_{1,1997}$=24.94, p<1.10^{-6}) than informed colonies using a fixed quorum rule.

When repeating the same analyses for each value of p_1 separately, we found the same results for p_1≤0.625 ($F_{1,397}$≥177.51, p<1.10^{-15} in all tests). However, colonies using the graded 'copy-when-uncertain' strategies were significantly more cohesive than informed colonies using a fixed quorum rule for p_1≥0.875 (p_1=0.875: $F_{1,397}$=163.30, p<1.10^{-15}; p_1=1: $F_{1,397}$=177.43, p<1.10^{-15}).

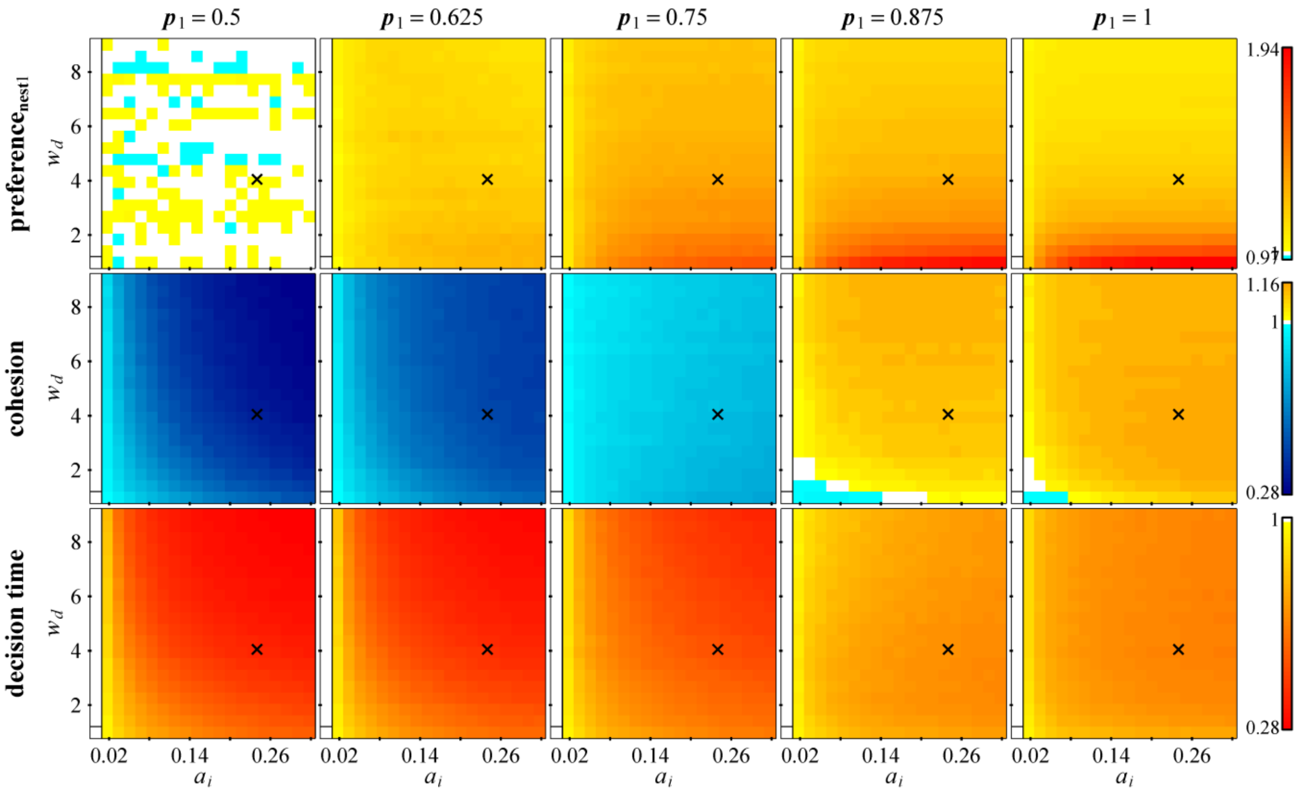

Figure S6. Comparisons of simulations for colonies using the 'copy-when-uncertain' strategy and informed colonies using a fixed quorum rule. Same as in main manuscript Figure 5. In order to facilitate visual comparisons between both strategies, heat maps now represent $\frac{value_{copy-when-uncertain}}{value_{fixed\ quorum rule}}$. For example, in the cohesion rows, all parameters values for which the plotted values are greater than 1 correspond to parameter values where colonies using the 'copy-when-uncertain' strategy were more cohesive than informed colonies using a fixed quorum rule.

II. Graded ‘copy-when-uncertain’ vs. naïve colonies

In order to facilitate data interpretation, we plotted the relative performance of colonies using the graded ‘copy-when-uncertain’ strategy compared to naïve colonies (p_i=0; Figure S7).

We used Student’s t-tests to test whether the accuracy, cohesion and emigration time of colonies using the graded ‘copy-when-uncertain’ strategy differed from the average value performance of naïve colonies. We found that overall, colonies using the graded ‘copy-when-uncertain’ strategies were faster (t = -64.47, df = 1899, p<1.10^{-15}), showed stronger preference for the majority nest (t = 117.23, df = 1519, p< 1.10^{-15}), but were less cohesive (t = -29.50, df = 1899, p<1.10^{-15}) than naïve colonies.

When repeating the same analyses for each value of p_1 separately, we found the same results for p_1≤0.75 (|t|≥29.10, df=379, p<1.10^{-15} in all tests). By contrast, colonies using the graded ‘copy-when-uncertain’ strategies were significantly more cohesive than naïve colonies for p_1≥0.875 (p_1=0.875: t = 39.17, df = 379, p<1.10^{-15}; p_1=1: t = 60.75, df = 379, p<1.10^{-15}).

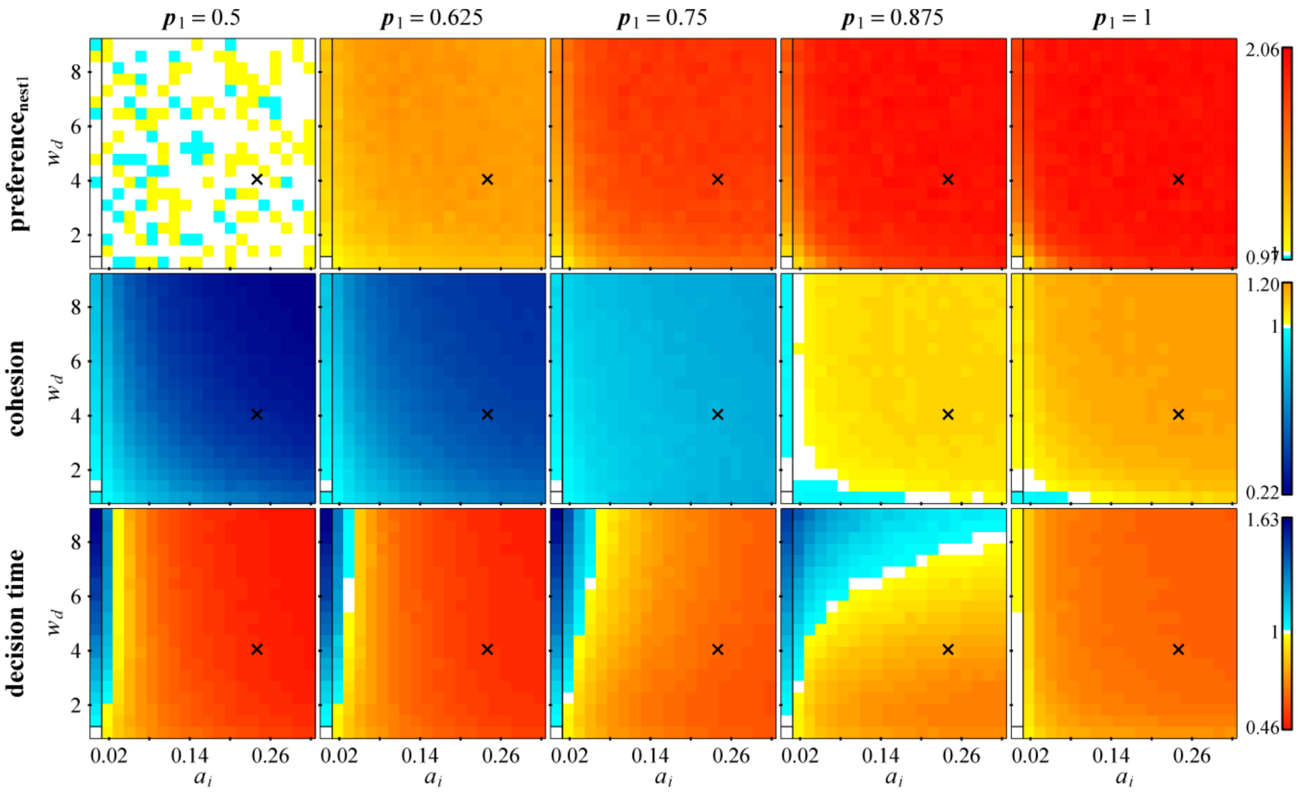

Figure S7. Comparisons of simulations for colonies using the ‘copy-when-uncertain’ strategy and naïve colonies. Same as in main manuscript Figure 5. In order to facilitate visual comparisons between both strategies, heatmaps now represent $\frac{value_{copy-when-uncertain}}{value_{naive}}$. For example, in the cohesion rows, all parameters values for which the plotted values are greater than 1 correspond to parameter values where colonies using the ‘copy-when-uncertain’ strategy were more cohesive than naïve colonies.

III. Graded 'copy-when-uncertain' vs. homogeneous informed behaviour

We repeated all simulations with parameter values p_{low}=0 and σ=1 (Figure S8; all other parameter values as in main manuscript). This corresponds to the case where all uninformed workers have a low probability of independent acceptance a_u, and all informed workers have the same probability of independently accepting the familiar nest $a_i \geq a_u$, regardless of the certainty of their private information. This differs from the graded 'copy-when-uncertain' strategy, where informed workers have varying probabilities of independently accepting the familiar nest depending on the certainty of their private information, with an overall average a_i. Figure S9 shows a point-by-point comparison of both strategies.

We used Wilcoxon matched-pairs tests to compare the simulation outcomes of both strategies for each $\{p_1, a_i, w_d\}$ triplet. We found that colonies using the 'copy-when-uncertain' strategies were slower (V = 1793500, $p<1.10^{-15}$), more cohesive (V = 1733500, $p<1.10^{-15}$) and showed stronger preference for the majority nest (V = 1018900, $p<1.10^{-15}$) than colonies using the alternative strategy.

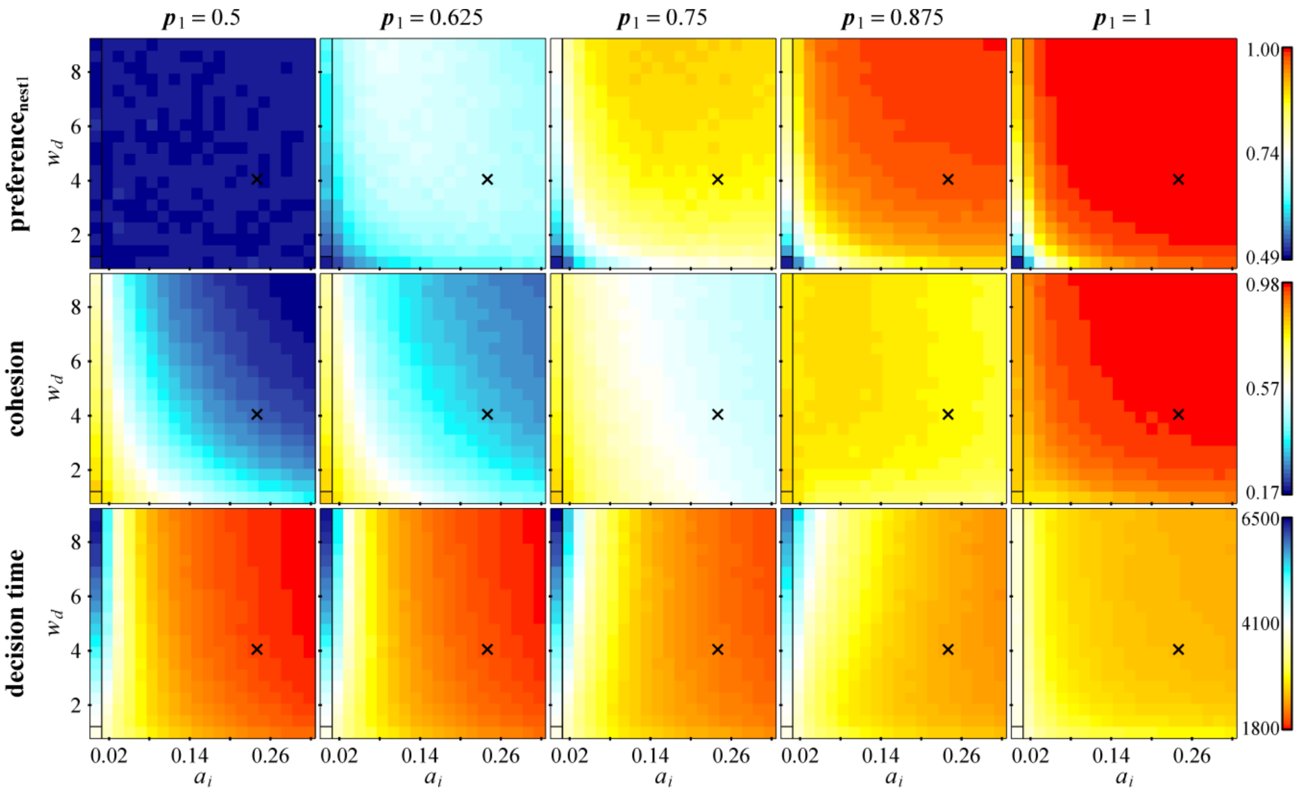

Figure S8. Simulation results for informed with homogeneous behaviour of informed workers. All representation conventions as in main manuscript Figure 5.

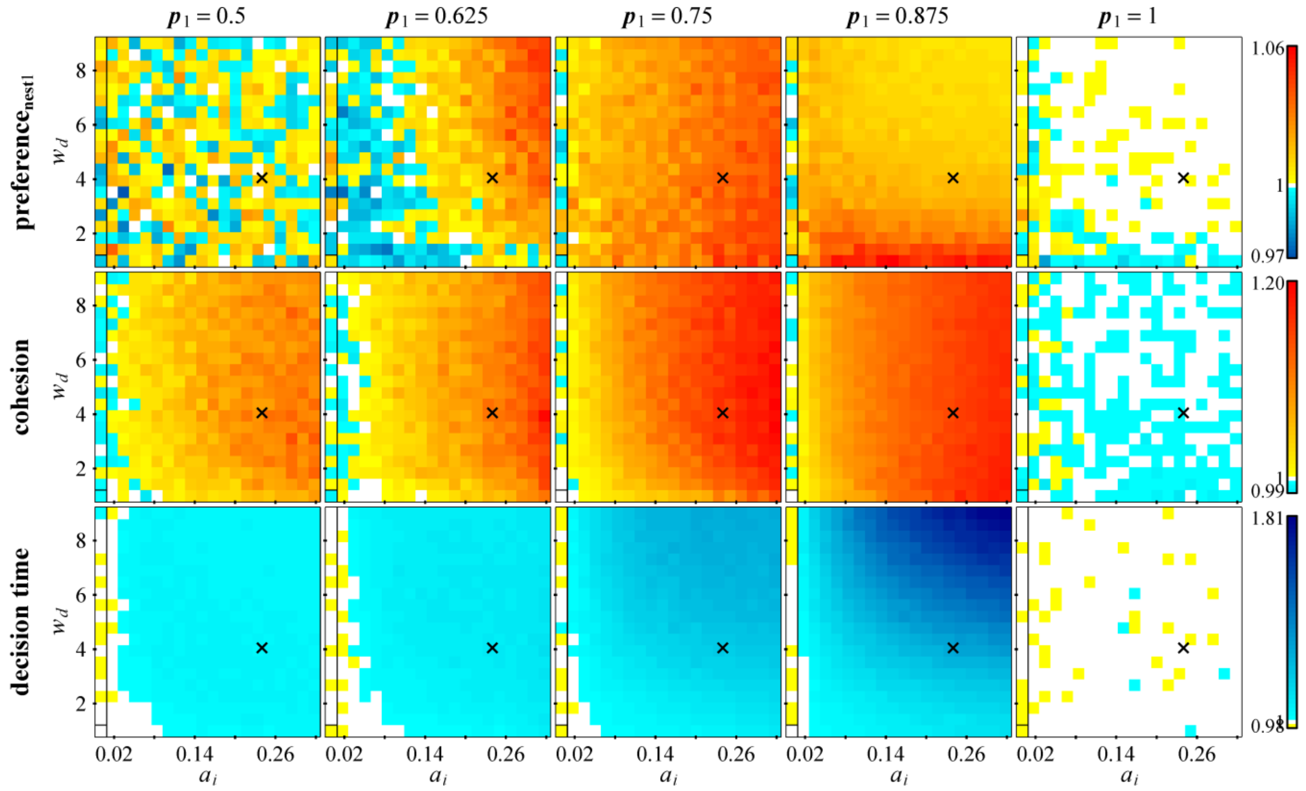

Figure S9. Comparisons of simulations for informed colonies using the 'copy-when-uncertain' strategy and informed colonies with homogeneous behaviour of informed workers. Same as in main manuscript Figure 5. In order to facilitate visual comparisons between both strategies, heatmaps now represent $\frac{value_{copy\text{–}when\text{–}uncertain}}{value_{homogeneous\ informed}}$. For example, in the cohesion rows, all parameters values for which the plotted values are greater than 1 correspond to parameter values where colonies where more cohesive when using the 'copy-when-uncertain' strategy than the alternative strategy.

Supplementary File 4
Influence of the different categories of workers on the decision process

1. Model simulations

The following analysis aimed at investigating the relative influence of different categories of workers (uninformed workers and informed workers with high, intermediate and low probability of independent acceptance) on the collective decision. For each combination of parameter values (see main manuscript), we performed 1,000 additional simulations where we recorded the order in which workers committed to each nest. The first few workers committing to each nest are those who influence the collective decision the most, because they increase the probability of their nestmates also committing to the nest: once the number of committed workers reaches the quorum threshold, all subsequent workers have a higher than 50% chance of themselves committing to the nest. We therefore calculated the proportion of workers of each category among the first workers committing to each nest until the quorum threshold was reached, or 'pre-quorum workers'. This was compared to the proportion expected under the null hypothesis of random commitment order, i.e. to the proportion of each category of workers among all workers modelled. Figure S10 shows the results obtained for the majority nest, N1, for the parameter values used in the main manuscript (n_i=24; p_{low}=0.5; σ=4; r =0; w_d ranging from 1 to 9 and a_i ranging from 0.1 to 0.31).

Relative to random expectations, informed workers were overrepresented among pre-quorum workers (except informed workers with low *a* for *ai* > 0.1). This overrepresentation increased with their probability of independent acceptance *a* (Figure S10 A). Informed workers with high *a* thus have the highest individual influence on the collective decision.

In addition, informed workers with high *a* were more numerous than any other worker category among pre-quorum workers at the majority nest provided a_i>0.05 (Figure S10 B). Informed workers with high *a* thus also have the highest influence as a group on the collective decision.

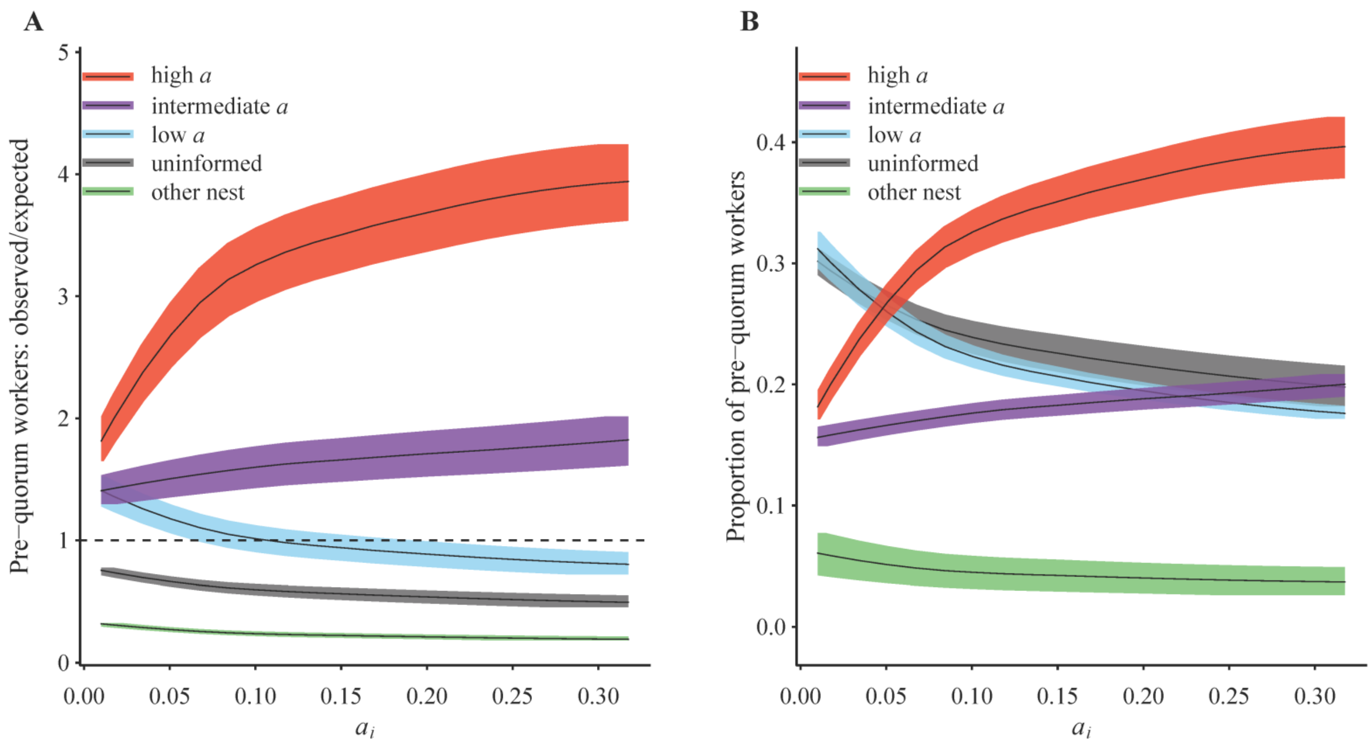

Figure S10. A. Relative weight of individuals from each worker category on the collective decision as a function of a_i. Lines and shading represent means and standard errors (calculated over all tested values of p_1 and w_d) of $\frac{observed\ proportion\ among\ pre\text{-}quorum\ workers\ at\ majority\ nest}{expected\ proportion\ among\ pre\text{-}quorum\ workers\ at\ majority\ nest}$ for each worker category. The hashed line represents expectations if observed values do not deviate from expectations, i.e. observed/expected=1. **B.** Proportion of workers from each category among pre-quorum workers at the majority nest as a function of a_i. Lines and shading represent means and standard errors (calculated over all tested values of p_1 and w_d) for each worker category.

2. *Experimental data*

We evaluated the relative contribution of the different categories of recruiters to the decision process in the experimental dataset from Stroeymeyt et al. 2011[1]. We counted the number of workers recruited to either nest by each category of recruiters (informed worker groups 1-4 and uninformed workers) during the early phase of the emigration, i.e. until the total number of recruits reached the quorum threshold. We used a chi-square test to compare these observed numbers to the expected numbers of recruits given the proportion of each category among all recruiters. Following Sharpe[2], we then evaluated which category of recruiters significantly deviated from null expectations by comparing the absolute value of the adjusted standardised residuals to a critical value z=2.58 (corresponding to a significance level of α=0.05/5 to account for multiple comparisons).

In agreement with the model prediction, we found the contribution of the 5 categories of recruiters to worker recruitment during the early deliberation process diverged significantly from random expectations (Chi-square test, $\chi2=60.55$, df=4, p<1.10-11). Residual analysis[2] showed that

this was due to informed workers from groups 1 and 2 recruiting significantly more workers than expected and uninformed workers recruiting significantly fewer workers than expected (group1: adjusted standardised residuals $\rho1=4.26$, group 2: $\rho2=5.01$; group 3: $\rho3=0.18$; group 4: $\rho4=-0.27$; uninformed: $\rho u=-6.63$; critical z-value for significance after adjustment for multiple comparisons: $|z|=2.58$ [2]; 73% of all recruitments directed towards the familiar nest). This confirms that informed workers with a non-zero probability of independent acceptance have a significantly higher influence on the deliberation process and the collective choice of the familiar nest than other workers.

References

1 Stroeymeyt, N., Franks, N. R. & Giurfa, M. Knowledgeable individuals lead collective decisions in ants. *J. Exp. Biol.* **214**, 3046-3054 (2011).

2 Sharpe, D. Your Chi-Square Test is Statistically Significant: Now What? . *Practical Assessment, Research & Evaluation* **20**, 1-10 (2015).

Supplementary File 5
Sliding-door system used to restrict access to the familiar nest

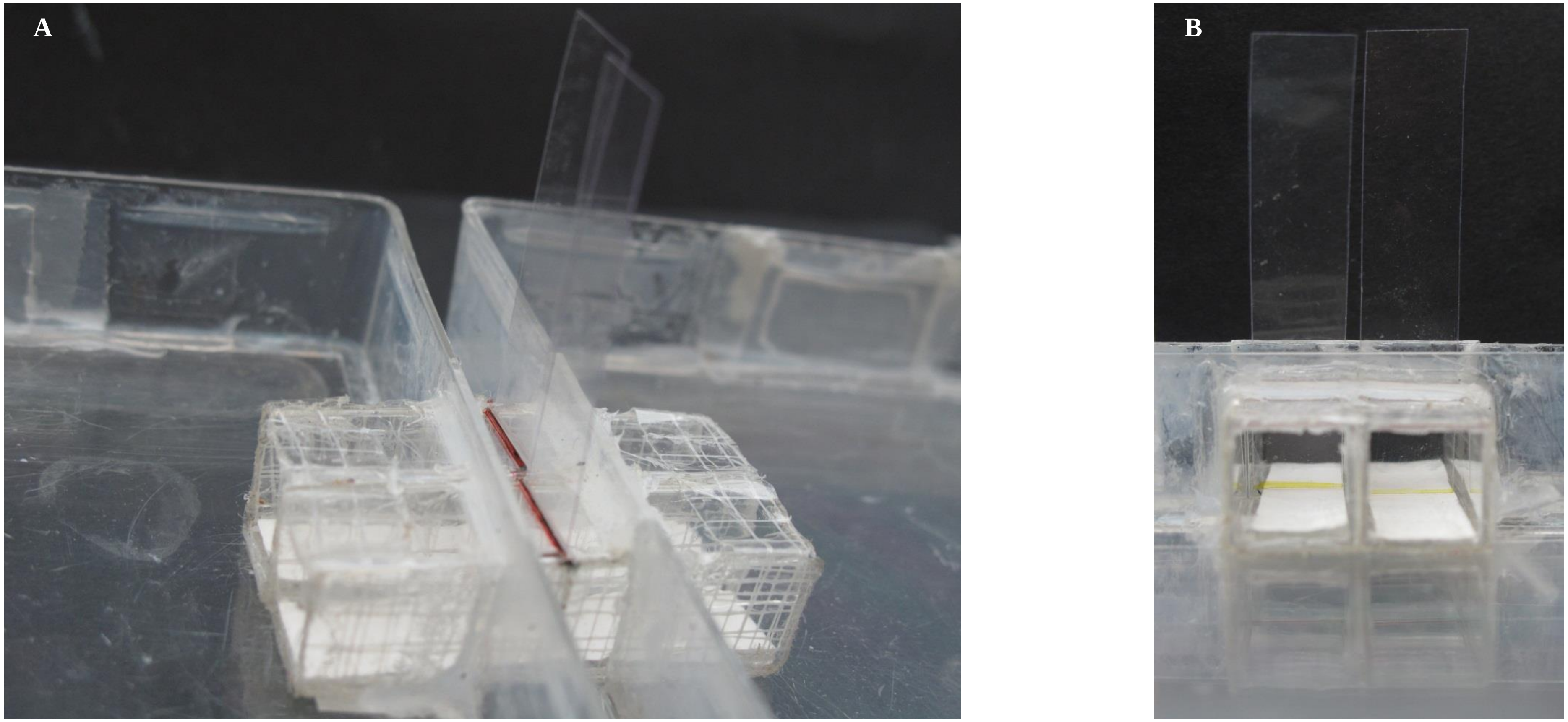

Side view (A) and front view (B) of the acetate sliding doors fitted through the tunnels leading to the peripheral dish containing the new nest. Sliding doors were introduced through a top slit cut in the middle of the tunnels and were lifted or lowered manually to allow or prevent passage.
